# Cytotoxic activity of crude extracts from *Datura stramonium*’s fungal endophytes against A549 lung carcinoma and UMG87 glioblastoma cell lines and LC-QTOF-MS/MS based metabolite profiling

**DOI:** 10.1186/s12906-019-2752-9

**Published:** 2019-11-21

**Authors:** Kudzanai Ian Tapfuma, Nkemdinma Uche-Okereafor, Tendani Edith Sebola, Raeesa Hussan, Lukhanyo Mekuto, Maya Mellisa Makatini, Ezekiel Green, Vuyo Mavumengwana

**Affiliations:** 1 Department of Biotechnology and Food Technology, Faculty of Science, University of Johannesburg, Box 17011, Doornfontein, Johannesburg, PO 2028 South Africa; 2 South African Medical Research Council Centre for Tuberculosis Research, Division of Molecular Biology and Human Genetics, Department of Biomedical Sciences, Stellenbosch University, Tygerberg, 7505 South Africa; 3 Department of Chemical Engineering, Faculty of Engineering and the Built Environment, University of Johannesburg, Box 17011, Doornfontein, Johannesburg, PO 2028 South Africa; 4 Molecular Sciences Institute, School of Chemistry, University of the Witwatersrand, P.O Box Wits, Johannesburg, 2050 South Africa

**Keywords:** *Datura stramonium*, Endophytes, Secondary metabolites, Lung carcinoma, Glioblastoma, Cytotoxicity

## Abstract

**Background:**

Endophytic fungi are a proven source of bioactive secondary metabolites that may provide lead compounds for novel drug discovery. In this study, crude extracts from fungal endophytes isolated from *Datura stramonium* were evaluated for cytotoxic activity on two human cancer cell lines.

**Methods:**

Fungal endophytes were isolated from surface sterilized aerial parts of *D. stramonium* and identified using molecular, morphological and phylogenetic methods. Ethyl acetate crude extracts from these isolates were evaluated for cytotoxic activity on A549 lung carcinoma and UMG87 glioblastoma cell lines. Metabolite profiling was then performed by liquid chromatography coupled to quadrupole time-of-flight with tandem mass spectrometry (LC-QTOF-MS/MS) for the cytotoxic crude extract.

**Results:**

Eleven fungal endophytes were identified from *D. stramonium*. Significant cytotoxicity was only observed from the crude extract of *Alternaria* sp. KTDL7 on UMG87 glioblastoma cells (IC_50_ = 21.49 μg/ml). Metabolite profiling of this crude extract tentatively revealed the presence of the following secondary metabolites: 1,8-dihydroxynaphthalene (1), anserinone B (2), phelligridin B (3), metacytofilin (4), phomopsidin (5) and vermixocin A (6). Compounds 2 and 3 have been shown to be cytotoxic in literature.

**Conclusion:**

The findings in this study suggest that the crude extract of *Alternaria* sp. KTDL7 possesses compound(s) cytotoxic to glioblastoma multiforme cells. Future studies to isolate and characterize the cytotoxic compound(s) from this fungus could result in lead development of a fungal-based drug for glioblastoma multiforme treatment.

## Background

Internal tissues of plants are habitats of a class of beneficial endosymbiotic microorganisms (predominantly bacteria and fungi) called endophytes that have been observed in all plants investigated to date [1]. In this plant-endophyte relationship, plants are hosts which generally offer nourishment and protection while endophytes improve plant defense, health and stress tolerance by solubilizing phosphates, fixing nitrogen, secreting siderophores, hydrolytic enzymes, antimicrobials or by producing plant hormones such as indole-3-acetic acid [2, 3].

In comparison to free living fungi, crude extracts of fungal endophytes are an underexplored but rich source of bioactive and chemically diverse secondary metabolites which include terpenoids, alkaloids, phenols, furandiones, dimeric anthrones and benzopyroanones [4, 5]. This is evidenced by a detailed review of 46 genera and 111 species of fungal endophytes producing cytotoxic secondary metabolites by Chen et al. [6]. In order to increase the likelihood of isolating fungal endophytes that produce medicinally important secondary metabolites, documented medicinal plants that are used in traditional medicine are targeted [5].

*Datura stramonium* is a medicinal plant that is known for producing over 64 tropane alkaloids of which atropine, scopolamine and hyoscyamine are predominantly found in relatively high concentrations [7, 8]. While ethnomedical uses of *D. stramonium* include inhalation of smoke from burnt leaves to relieve symptoms of asthma, bronchitis, sedation, epilepsy and psychosis to name just a few [8], exploration into the use of tropane alkaloids as potentially anticancer lead compounds has been ongoing since the early 2000s [9]. Bacterial and fungal endophytes have been previously isolated from *D. stramonium* in studies focusing on the potential use of endophytic extracts as biocontrol agents for controlling plant and human pathogens [10–13], in vitro α-glucosidase inhibitors and antioxidant agents [14]. To the best of our knowledge, this is the first study that reports the cytotoxic activity of crude extracts endophytic fungi from *D. stramonium* on human A549 lung carcinoma and UMG87 glioblastoma cell lines. The results of the bioactive crude extract observed in this study may form a foundation for developing a fungal-derived drug for glioblastoma multiforme treatment.

## Methods

### Collection of plant material

Healthy free growing *D. stramonium* plants were collected in summer in Johannesburg (South Africa) at the following coordinates: 26°13′04.5″S, 28°12′48.3″E. Plant diversity and vegetative growth on the site were high with different species interspersed between *D. stramonium*. Plant samples were transferred to the laboratory immediately after collection and were thoroughly washed with distilled water upon arrival. Formal identification of the collected plants was done by Abdulwakeel Ayokun-nun Ajao, a botanist from the Department of Botany and Plant Biotechnology at the University of Johannesburg. A voucher specimen of the whole plant was deposited in the department’s public herbarium and was assigned deposition number RAM-001.

### Isolation and morphological characterization of fungal endophytes

The isolation of fungal endophytes was done on the same day of collection following a modified method described by Uche-Okereafor et al., [15]. Briefly, 10 g of each of the aerial plant parts (stems, leaves, fruit covers and seeds) were separately soaked in 5% Tween 80, adequate to cover each sample for five minutes with vigorous shaking. This was subsequently followed by washing the samples several times with sterile distilled water to remove Tween 80. Samples were then dipped in 70% ethanol for 1 min and rinsed with sterile distilled water five times, followed by dipping in 1% sodium hypochlorite for 10 min. Plant parts were then finally rinsed five times with sterile distilled water and aliquots of 50 μL of the last rinse water for each sample were plated on potato dextrose agar (PDA) (Merck, Johannesburg, SA) as wash controls to determine the effectiveness of surface sterilization. The surface sterilized samples were then macerated in sterile phosphate buffered saline (PBS) (Oxoid, Basingstoke, Hampshire, UK) solution using a sterile mortar and pestle. Serial dilutions of macerated samples were made by pipetting 1 mL of macerated sample into 9 mL of PBS to make a 10^− 1^ dilution, followed by subsequent dilutions up to 10^− 9^. The serial dilutions were then plated in triplicates on PDA for enumeration of fungal endophytes and incubated at 28 °C (IncoTherm, Labotec, Johannesburg, SA) for up to 21 days. Morphologically distinct fungal isolates were then sub-cultured several times to obtain pure isolates. Fungi were differentiated from bacteria using lactophenol cotton blue staining. Lactophenol cotton blue is a dye which stains chitin in fungal cell walls blue [16].

### Molecular characterization (rDNA-ITS sequencing and phylogenetic analysis)

DNA extraction was done using the ZR Fungal/Bacterial DNA Kit™ (Zymo Research, Irvine, CA, USA), following the manufacturer’s instructions. Polymerase chain reaction (PCR) was done to amplify the internal transcribed spacer (ITS) region of ribosomal DNA (rDNA) using the ITS1 (5´-TCCGTAGGTGAACCTGCGG-3´) and ITS4 (5´-TCCTCCGCTTATTGATATGC-3´) primer pair. Forward and reverse direction sequencing was done using the ABI PRISM™ 3500xl Genetic Analyzer (Thermo Fisher Scientific, Inc., Waltham, MA, USA) followed by the purification of the sequencing products using ZR-96 DNA Sequencing Clean-up Kit™ (Zymo Research, Irvine, CA, USA). DNA sequences were then analyzed using the FinchTV software [17], followed by a Nucleotide Basic Local Alignment Search (BLASTN) on the National Center for Biotechnology Information (NCBI) using the GenBank database to identify closely matching organisms [18]. The sequences used in the molecular data sets ranged from 450 to 700 base pairs prior to deletion of ambiguous data occurring at the beginning or at the end of each sequence [19]. Maximum likelihood phylogenetic reconstruction was done using MEGA version 7.0 software [20], with *Dothidea insculpta* and *Monochaetia monochaeta* as outgroups. Bootstrap values were calculated from 1000 replicate runs. Phylogenetic reconstruction of isolates was done by grouping isolates according to morphological characteristics observed on PDA cultures. The rDNA-ITS sequences were then submitted to GenBank.

### Shannon-Weiner diversity index (*H´*)

Fungal endophyte diversity was determined by the Shannon-Wiener diversity index (*H´*), using the formulae below:

$$ H\acute{\mkern6mu}=\varSigma \left( Pi\times \mathit{\ln}\  Pi\right), Pi=\frac{mi}{N.} $$

where mi represents number of individuals and N represents the total number of individuals [21].

### Fermentation and extraction of secondary metabolites

Fungal endophytes were fermented as monocultures in 3 L of PDB (Potato infusion 200 g/L, dextrose 20 g/L) [22]. Incubation was done for 21 days at 28 °C in an orbital shaking incubator (Amerex Gyromax, Temecula, CA, USA) at 150 rpm. After fermenting the fungi, extrolites which are mainly secondary metabolites were extracted from broth monocultures using analytical grade ethyl acetate [23]. This extraction was achieved by firstly filtering the broth monocultures through a Whatman No. 1 filter paper to separate the mycelia from the broth culture. Equal volumes of ethyl acetate and filtrate broth were then added to a separating funnel, shook vigorously to mix the two liquids and allowed to stand for 20 min. The organic solvent phase was then collected and concentrated using a rotary evaporator under reduced pressure at 40 °C and the resulting crude extracts were allowed to air dry and consequently stored at − 20 °C.

### MTS assay on UMG87 glioblastoma and A549 lung carcinoma cell lines

End-point cytotoxicity evaluation of crude extracts on UMG87 glioblastoma and A549 lung carcinoma cell lines (ATCC, Manassas, VA, USA) was performed following the colorimetric MTS [3-(4,5-dimethylthiazol-2-yl)-5-(3-carboxymethoxyphenyl)-2-(4-sulfophenyl)-2*H*-tetrazolium] assay method [6, 10]. Cells at 5 × 10^4^ cells/mL were initially seeded in 96 well plates containing Dulbecco’s modified eagle medium (Gibco, Carlsbad, CA, USA) with 15% heat inactivated fetal bovine serum (Merck, Johannesburg, SA) and incubated at 37 °C in 5% CO_2_ (v/v) for 24 h [24]. Crude fungal extracts and auranofin (a positive control) were then dissolved in dimethyl sulfoxide (DMSO) (Merck, Johannesburg, SA) and then added to cell cultures at concentrations of 3.13, 6.25, 12.5, 25, 50 and 100 μg/mL, in triplicates. The cell cultures were then left to incubate for a further 96 h, after which 5 μl of MTS (Promega, Madison, WI, USA) was added to the cells and absorbance values measured at 490 nm after 1, 2 and 4-h incubation periods. Cell viability was calculated using the following formulae:

$$ \%\mathrm{Cell}\ \mathrm{Viability}=\frac{{\mathrm{E}}_{\mathrm{a}}-{\mathrm{B}}_{\mathrm{a}}}{{\mathrm{C}}_{\mathrm{a}}-{\mathrm{B}}_{\mathrm{a}}}\times 100 $$

where E_a_ is absorbance of the extract, B_a_ is absorbance of the blank and C_a_ is the absorbance of the negative control (untreated cells) [25]. GraphPad Prism software (v. 7.05, GraphPad Software, Inc., La Jolla, CA, USA) was used to produce dose response curves by non-linear regression analysis of cell viability data, hence determining the mean inhibitory concentration (IC_50_) value.

### xCELLigence® real-time cell analyzer (RTCA) assay on U87MG glioblastoma cells

xCELLigence® RTCA assay was performed by initially seeding 1 × 10^5^ cells/mL of U87MG glioblastoma cells on gold microelectrode precoated 96 well electronic plates (E-Plate® 96, ACEA Biosciences Inc., San Diego, CA, USA) and incubating at 37 °C in 5% CO_2_ (v/v) for 45 h. Selected crude fungal extracts and auranofin (a positive control) were then dissolved in DMSO and then added at concentrations of 3.13, 6.25, 12.5, 25, 50 and 100 μg/mL, in triplicates. Untreated cells (0 μg/mL) were included as a negative control. The cell cultures were then incubated for a further 171 h, with impedance measurements taken every 15 min during the total incubation period of 216 h. The data was retrieved, and a graphic representation of the bioactivity was reproduced.

### Metabolite profiling of fungal crude extracts by LC-QTOF-MS/MS

Metabolite profiling of the cytotoxic fungal extract was done by liquid chromatography coupled to a quadrupole time-of-flight with tandem mass spectrometry (LC-QTOF-MS/MS), using a previously described modified method [26, 27]. This system has a Dionex UltiMate 3000 ultra-high-performance liquid chromatography (UHPLC) (Thermo Scientific, Darmstadt, Germany) coupled to a Compact™ QTOF (Bruker Daltonics, Bremen, Germany) that uses an electrospray ionization (ESI) interface. The crude extract of the fungal endophyte *Alternaria* sp. KTDL7 was prepared for analysis by dissolving 1 mg/mL (w/v) in HPLC grade methanol (Merck, Johannesburg, SA), followed by sonication for 10 min, and finally filtration through 0.22 μm polyvinylidene fluoride (PVDF) membrane syringe filters into 1 mL LC auto-sampler vials. An injection volume of 5 μL was used in the system for chromatographic separation of analytes in reverse phase ultra-high-performance liquid chromatography (RP-UHPLC) through a Raptor ARC-18 column with dimensions of 2.7 μm (particle size), 2.1 mm (internal diameter), 100 mm (length) and 90 Å (pore size) (Restek, Bellefonte, PA, USA). The mobile phase was composed of solvent A (A) consisting of 0.1% formic acid in H_2_O (v/v) and solvent B (B) consisting of 0.1% formic acid in acetonitrile (v/v). Gradient flow of the mobile phase was initiated by a 2.0 min isocratic step at 5% B followed by an increase to 95% in 28 min, an isocratic step at 95% B for 5 min followed by a decrease to 5% B in 1 min upon re-equilibration to initial conditions at a flow rate of 300 μL/min. The ESI(+) parameters were as follows: set capillary voltage at 4.5 kV; end plate offset at − 500 V; dry heater temperature at 220 °C; dry gas flow rate at 2.5 L/min and nebulizer gas pressure at 1.8 Bar. Mass spectra were acquired in centroid mode ranging from 50 to 1300 *m/z* [28]. Instrument operation, control and data acquisition was done using HyStar software version 2.10 (Thermo Scientific, Darmstadt, Germany). Spectral data processing was performed in Bruker Compass DataAnalysis software version 4.3 (Bruker Daltonics, Bremen, Germany). MetFrag web tool version 2.1 (https://msbi.ipb-halle.de/MetFragBeta/) was used to characterize the resulting fragment spectra by linking to three compound databases, namely PubChem, ChemSpider and KEGG [29]. The MetFrag settings used were as follows: The MetFrag database search settings used were as follows: Database search relative mass deviation (Search ppm) = 10.0; precursor ion = [M + H]^+^; fragment peak match absolute mass deviation (Mzabs) = 0.01; fragment peak match relative mass deviation (Mzppm) = 10; charge = positive and mode = [M + H]^+^.

### Statistical analysis

Quantitative variables were analyzed in STATISTICA version 10 (StatSoft, Inc., Tulsa, OK, USA). Multivariate analysis of variance (MANOVA) and the least significant difference (LSD) post hoc were used to analyze the mean ± standard deviation (SD) of crude extracts at various concentrations. A probability of *P* ≤ 0.05 was taken to indicate statistical significance.

## Results

### Isolation, characterization and identification of culturable fungal endophytes

In this study, 11 culturable fungal endophytes were recovered from *D. stramonium* (seven isolates from the leaves, three from the stems and one from the seeds). Examination of morphological macroscopic and microscopic features revealed that four out of eleven were filamentous fungi. Analysis of the ITS sequences resulted in the taxonomic classification of five fungal isolates to species level with the rest only classified to genus level (Table 1). These results corroborated with the phylogenetic reconstruction which grouped isolates according their respective genera and species (see Additional file 1). The Shannon-Weiner diversity index (*H´*) for the isolated endophytes was calculated and found to be 3.44 with the highest diversity observed in isolates from the leaves. This diversity index takes into account homogeneity/heterogeneity of isolates and usually ranges between 1.5 to 4.5, where the higher values correspond to increase in species diversity [30].

**Table 1:** Eleven fungal endophytes isolated from *D. stramonium*

Fungal isolate	Accession number	Closest relatives in NCBI	ITS identity (%)	Tissue	Phylum; Class; Order	Classification
KTDL1	MF952612	*Gyroporus castaneus* Gc1 (EU718099)	88	Leaves	Basidiomycota; Agaricomycetes; Boletales	*Gyroporus* sp*.*
KTDL2	MF952613	*Alternaria tenuissima* Isolate 4 (KU937315)	97	Leaves	Ascomycota; Dothideomycetes; Pleosporales	*A. tenuissima*
KTDL3	MF952614	*A. alternata* CS36–4 (KY814634)	100	Leaves	Ascomycota; Dothideomycetes; Pleosporales	*A. alternata*
KTDL4	MF952615	*Colletotrichum* sp. LTL119 (MF663557)	100	Leaves	Ascomycota; Sordariomycetes; Glomerellales	*Colletotrichum* sp.
KTDL6	MF952616	*Talaromyces* sp*.* SWP-2017 k NRRL 62271 (KX657354)	89	Leaves	Ascomycota; Eurotiomycetes; Eurotiales	*Talaromyces* sp*.*
KTDL7	MF952617	*Alternaria* sp. XN-3-1 (KR822138)	100	Leaves	Ascomycota; Dothideomycetes; Pleosporales	*Alternaria* sp.
KTDL8	MF952618	*Sporothrix schenckii* CBS 211.61 (KP017093)	100	Leaves	Ascomycota; Sordariomycetes; Ophiostomatales	*Sporothrix schenckii*
KTDL11	MF952619	*Trichoderma longibrachiatum* FIB PRI 6.2 (LC106115)	91	Seeds	Ascomycota; Sordariomycetes; Hypocreales	*Trichoderma sp*.
KTDS1	MF952620	*Pilobolus crystallinus* 007pNNP (KP760865)	98	Stem	Zygomycota; Mucoromycotina; Mucorales	*Pilobolus crystallinus*
KTDS2	MF952621	*Rhodotorula mucilaginosa* Feni 103 (KP223714)	99	Stem	Basidiomycota; Urediniomycetes; Sporidiales	*Rhodotorula mucilaginosa*
KTDS5	MF952622	*Bipolaris setariae* GP14 (KR183790)	99	Stem	Ascomycota; Dothideomycetes; Pleosporales	*Bipolaris* sp*.*

### MTS cytotoxicity assay on A549 lung carcinoma cells

**Fig. 1:**
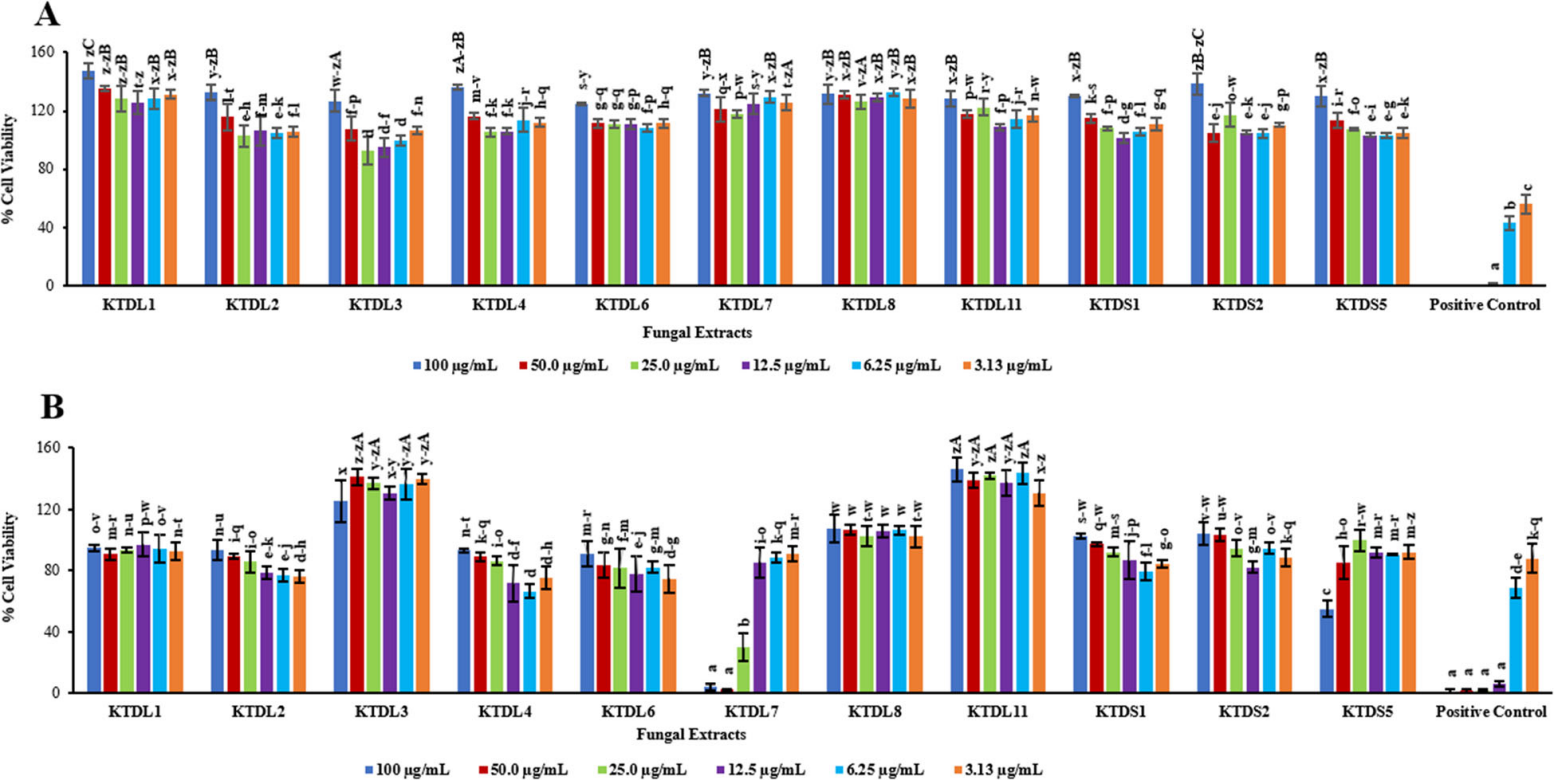
A summative profile of bioactivity activity of fungal extracts on (**a**) A549 lung carcinoma cells and (**b**) UMG87 glioblastoma cells after 96 h of exposure. Columns in the histograms represent the mean ± SD (*n* = 3) of fungal crude extracts tested at six different concentrations ranging from 3.13 to 100 μg/mL. The positive control was auranofin and the alphabets above the columns represent significant differences among various concentrations of extracts

Statistically significant differences in the effect of fungal crude extracts on A549 lung carcinoma cells were observed at *P* ≤ 0.05 level even though the cytotoxicity observed was limited. Cell viability ranged from 92.2 to 146.9%, reflecting limited inhibitory effect presented by the fungal crude extracts during the incubation period of 96 h (Fig. 1). Cell viability of above 100% was mostly observed for the highest concentrations of fungal crude extracts (25, 50 and 100 μg/mL), which may typically have resulted from the antioxidant potential of compounds in fungal crude extracts, causing elevated absorbance values for the reduced of MTS product (formazan) that are higher than those observed in the negative control cells [31, 32].

### MTS cytotoxicity assay on UMG87 glioblastoma cells

**Fig. 2:**
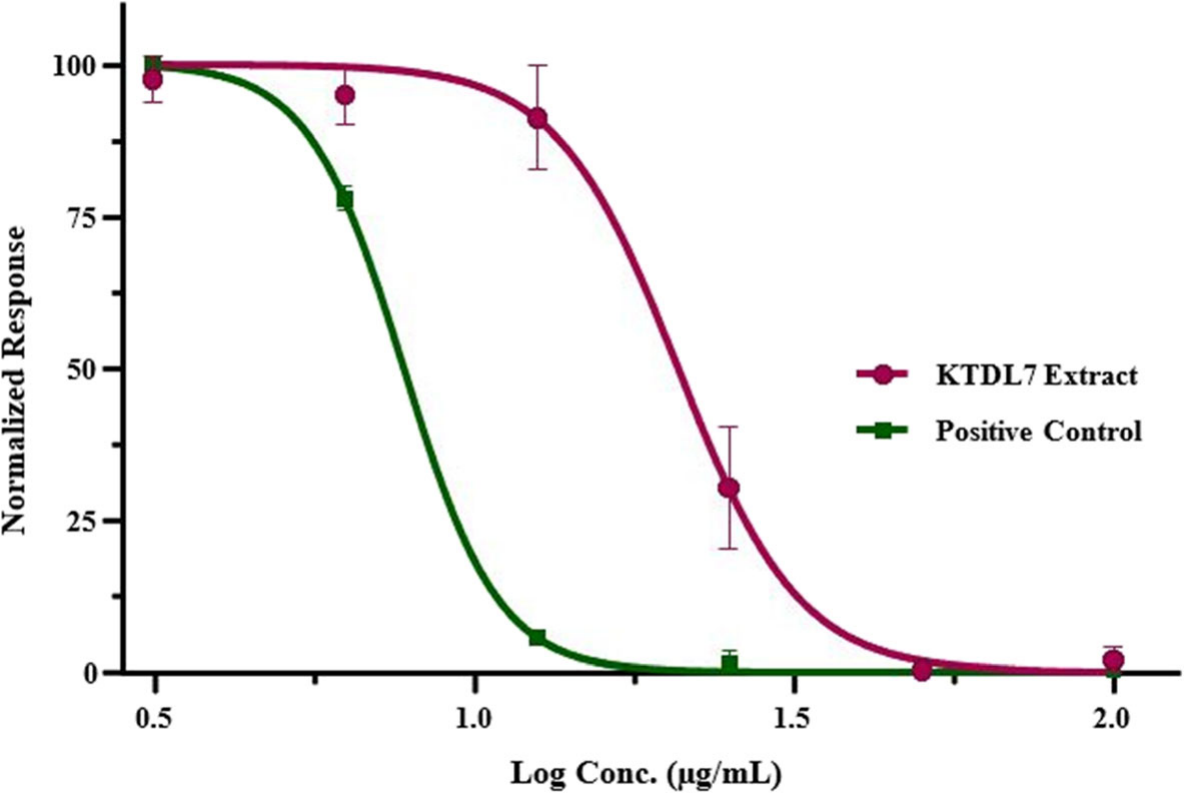
Dose-response inhibition curve of the crude extract of *Alternaria* sp. KTDL7 on UMG87 glioblastoma cells

The crude fungal extract from *Alternaria* sp. KTDL7 showed the highest antiproliferative activity on UMG87 glioblastoma cells, recording the lowest cell viability of 2.68% at 50 μg/mL, followed by 4.29% at 100 μg/mL (Fig. 1). Multi-variate analysis of variance test of the means from the two concentrations showed that their cytotoxic activity had no significant difference since *P >* 0.05. Furthermore, the cytotoxic effects of these two concentrations from *Alternaria* sp. KTDL3 were found to be comparable with that of auranofin on the same cell line at treatments of 12.5–100 μg/mL (Fig. 1). The IC_50_ value for the fungal extract from *Alternaria* sp. KTDL7 was determined by plotting a dose-response curve (Fig. 2) and was found to be 21.49 μg/mL, just below the American National Cancer Institute guidelines (NIC) for preliminary screening assays which state that crude extracts achieving 50% anti-proliferative activity at < 30 μg/mL after 72 h of exposure are to be regarded as cytotoxic [33, 34].

### xCELLigence® RTCA assay on UMG87 glioblastoma cells

**Fig. 3:**
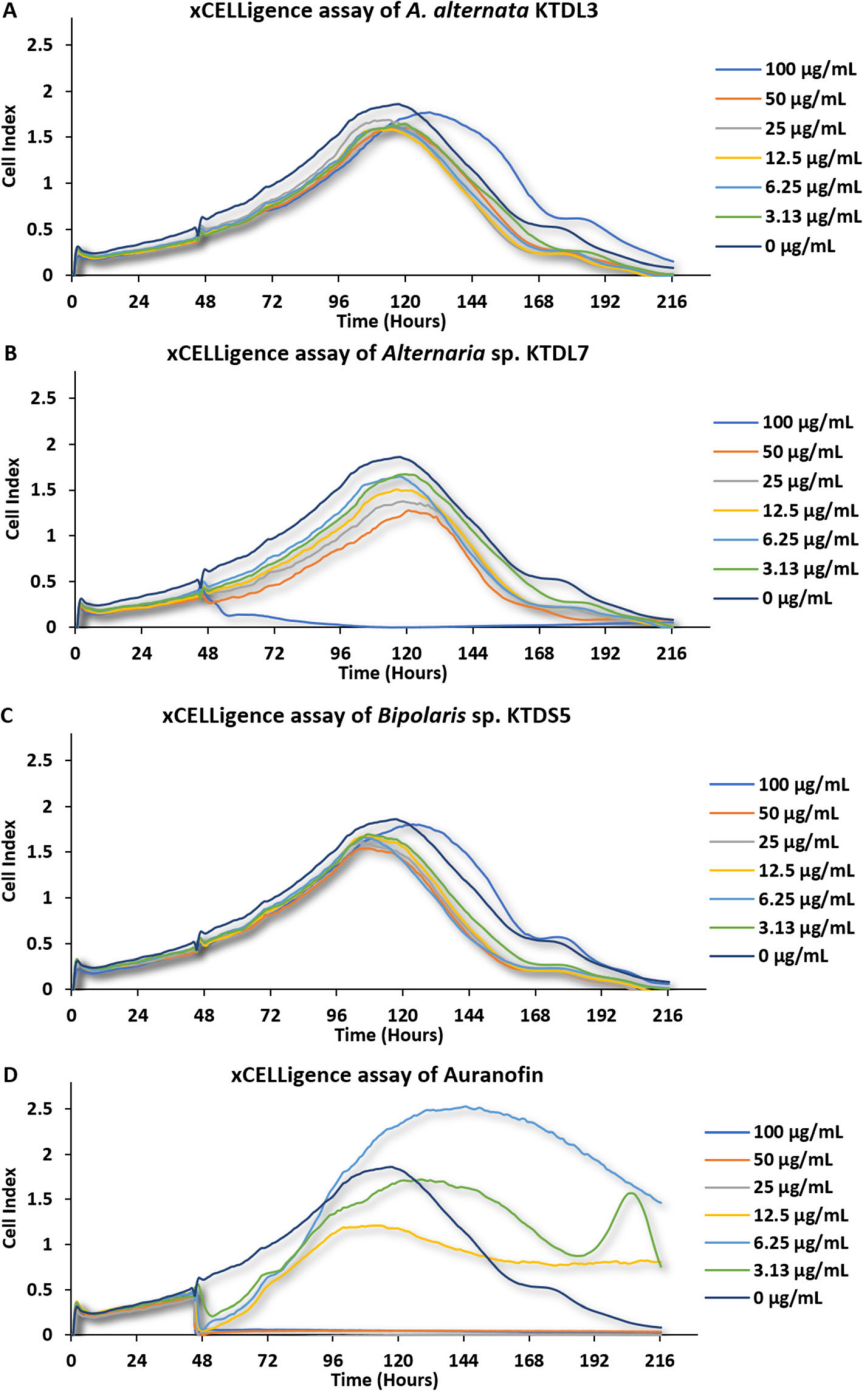
Real-time analysis of the bioactivity of crude fungal extracts on UMG87 glioblastoma cells. Extracts from *Alternaria alternata* KTDL3 (**a**), *Alternaria* sp. KTDL7 (**b**), and *Bipolaris* sp. KTDS5 (**c**) were administered in six concentrations ranging from 0 to 100 μg/mL on the 45th hour on the timeline and the response of the cells was monitored up until the 216th hour. Cell viability was recorded as cell index, which is a relative change in measured impedance. Auranofin (**d**) was used as a positive control

UMG87 glioblastoma cells were exposed to selected fungal crude extracts *of A. alternata* KTDL3, *Bipolaris* sp. KTDS5 and *Alternaria* sp. KTDL7 which was initially observed to induce cytotoxicity on this cell line. Response of the cells to the fungal extracts was monitored for 171 h using a RTCA system. A dose-dependent inhibition was observed for the crude extract of *Alternaria* sp. KTDL7, where the highest concentration of 100 μg/mL induced an irreversible cytotoxic effect on the UMG87 glioblastoma cells as shown in Fig. 3. Cells exposed to 100 μg/mL of the crude extract were unable show significant recovery from the cytotoxic effects from the point of exposure at the 45th hour to the 216th hour on the timeline.

Auranofin (a positive control) had a striking effect on UMG87 glioblastoma cells as some concentrations seemed to promote hyper metabolism than inhibit it. Exposure of the cells to a drug concentration of 6.25 μg/mL at the 45th hour resulted in an immediate decline in cell viability followed by a recovery and surge in viability from the 96th hour which exceeded the cell viability of the negative control (0 μg/mL) and cells treated with 3.13 μg/mL of the drug. The surge in viability of cells treated with 6.25 μg/mL of auranofin at the 96th hour could be associated with development of antineoplastic resistance of surviving cells, leading them to overcome the cytotoxic effects of auranofin by upregulation of metabolic genes and thus leading to a spike in cell viability. Mechanisms of drug resistance in glioblastoma cells have been reviewed in Haar et al., [35]. Similar to the MTS assay, no significant cytotoxic activity was observed for the crude extracts of *A. alternata* KTDL3 and *Bipolaris* sp. KTDS5.

### Metabolite profiling of *Alternaria* sp. KTDL7’s crude extract by LC-QTOF-MS/MS

**Fig. 4:**
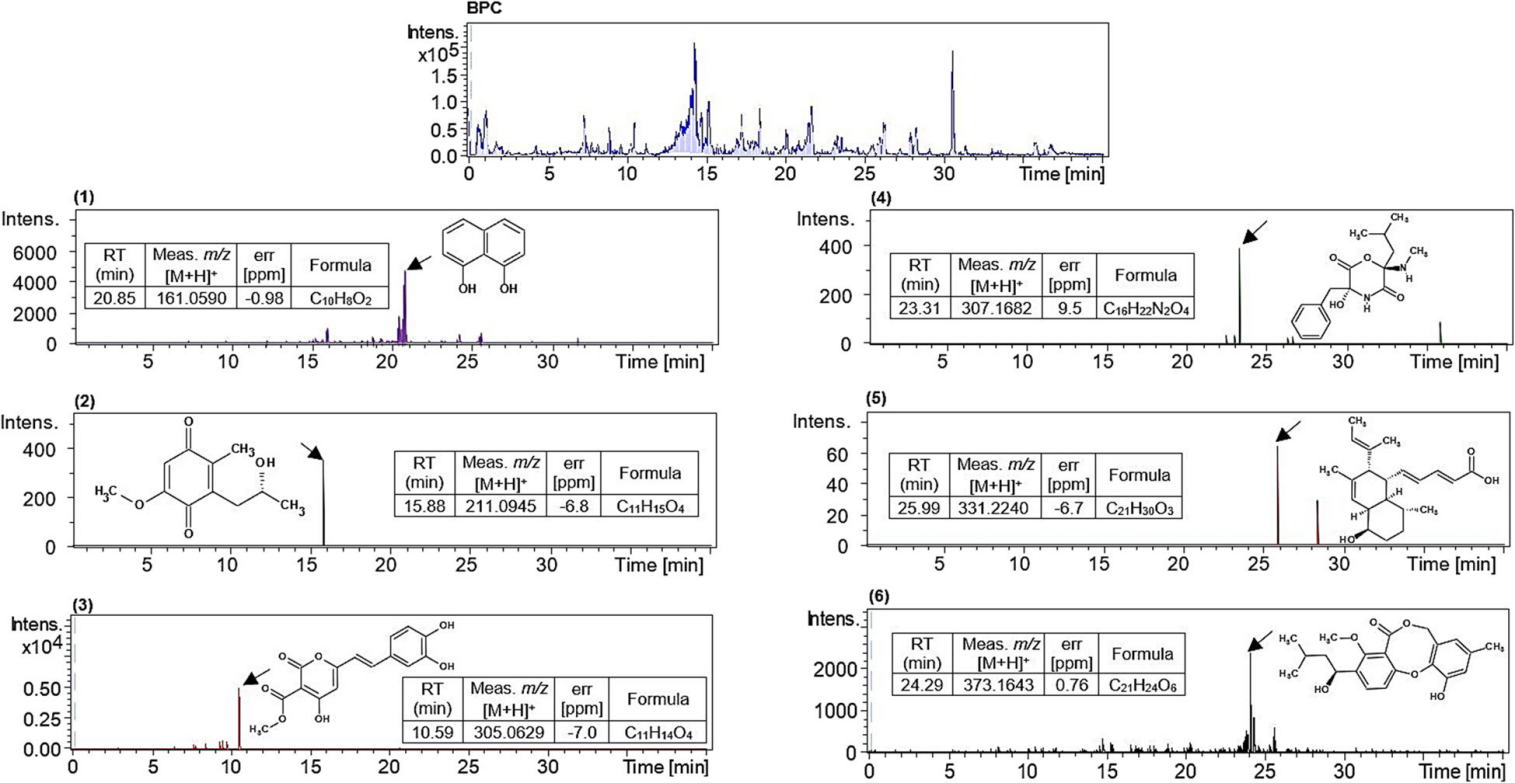
The base peak chromatogram (**bpc)** of *Alternaria* sp. KTDL7’s crude extract and the extracted ion chromatogram (EIC) of identified secondary metabolites which are: 1,8-dihydroxynaphthalene (**1**), anserinone B (**2**), phelligridin B (**3**), metacytofilin (**4**), phomopsidin (**5**) and vermixocin A (**6)**. Meas. *m/z* denotes measured *m/z*, while Calc. *m/z* denotes calculated *m/z*

Secondary metabolites in the crude extract of *Alternaria* sp. KTDL7 were tentatively identified using an untargeted screening method. The impact of PDB on the fungal crude extract was considered by analyzing the spectrum of PDB and subtracting it from the spectrum of the fungal crude extract. Secondary metabolites were identified using the spectral information of molecular ions and their collision induced dissociation (CID) fragments which were compared with reference compounds and their in-silico fragments in online databases (Fig. 4) [36]. The identified compounds are as follows: 1,8-dihydroxynaphthalene (**1**), anserinone B (**2**), phelligridin B (**3**), metacytofilin (**4**), phomopsidin (**5**) and vermixocin A (**6**). CID mass fragment data is available in Additional file 2.

## Discussion

Medicinal plants with known ethnopharmacological properties are proven sources for isolation of endophytes that produce secondary metabolites with novel and medically significant bioactivities [21, 37]. The surface sterilization method of isolating endophytes is highly effective to reduce contamination of epiphytes when sodium hypochlorite is employed [38]. In this study, efficacy of surface sterilization was validated by plating on PDA the last rinse water used in the surface sterilization process as a control. No microbial growth was observed on these plates.

The Shannon-Wiener diversity index (*H´*) for the isolated endophytes was calculated and found to be 3.44, indicating a high species diversity among the fungal endophyte community in *D. stramonium*. Greatest diversity was observed in the leaves where the highest number of isolates were recovered with the *Alternaria* genus being the most prevalent. This genus has been previously reported as an endophyte in *D. stramonium* [39], while also being a pathogen in other plants of a different species which include cereals, strawberries and tomatoes [40]. Interestingly, pathotypes of the *Alternaria* genus mostly occur as foliar pathogens which produce host-selective toxins (HSTs) to target the above-mentioned susceptible plants [41]. Both the endophytes and pathotypes of this genus are rarely isolated from the seeds and roots, and less frequently from the stems [40–43].

The three endophytes from the *Alternaria* genus (*A. tenuissima* KTDL2, *A. alternata* KTDL3 and *Alternaria* sp. KTDL7) produced varying shades of dark brown pigmented hyphae due to melanin production, a pigment known to improve stress tolerance of plant hosts by trapping and eliminating oxygen radicals generated during abiotic stress [44]. The endophyte *Bipolaris* sp. KTDS5 appeared to have a mixture of black pigmented and nonpigmented white colonies, where the black pigment was also evidence of fungal melanin production [45]. Endophytes that produce melanized septate hyphae and microsclerotia-like structures are commonly known as “Dark Septate Endophytes” (DSE), they are collectively thought to improve nutrient acquisition and stress tolerance in plants [46]. *R. mucilaginosa* KTDS2 had pink colonies and *G. castaneus* KTDL1, *Colletotrichum* sp. KTDL4, *Talaromyces* sp. KTDL6, *S. schenckii* KTDL8, *Trichoderma* sp. KTDL11 and *P. crystallinus* KTDS1 all had a cream-white appearance. Pigments in fungi are chiefly produced in the mevalonate pathway and include carotenoids such as lycopene, γ-carotene, β-carotene, cantaxanthin, astaxanthin, neurosporaxanthin and torulene [47]. Besides contributing to the metabolism of the host plant, natural pigments produced by (endophytic) fungi have great potential in the food and beverage industry where synthetic pigments are often toxic and carcinogenic [48]. The specific individual roles played by each fungal isolate in the plant-endophyte relationship with *D. stramonium* are still yet to be better understood.

Among the isolated fungal endophytes in this study, significant and selective cytotoxic activity was observed from the crude extract of *Alternaria* sp. KTDL7 on UMG87 glioblastoma cells in the MTS assay. The highest cytotoxic activity of this crude extract was observed at 50 and 100 μg/mL, indicating a dose-response dependent activity. Still on the same fungal extract and cell line, an interesting observation was noted whereby the actual cell viability of the 50 μg/mL treatment (2.68%) was found to be 1.61% lower than that of double the concentration, the 100 μg/mL (4.29%) treatment (Fig. 1). Upon testing the two means with multivariate analysis of variance, no significant statistical difference in their activity was found as the *p*-value *P* > 0.05. Mechanisms underlying the selective cytotoxicity observed from the crude extract of *Alternaria* sp. KTDL7 were not investigated as this was beyond the scope of this study.

In the xCELLigence assay, the cytotoxic activity of the crude extract of *Alternaria* sp. KTDL7 at 100 μg/mL on UMG87 glioblastoma cells was observed to be much higher and not comparable to that of the same extract at 50 μg/mL (Fig. 3). The resulting differences in the behavior of this extract when assayed in the xCELLigence and MTS assay can be explained by the fact that both assays target different markers. The xCELLigence assay determines cell viability indirectly by measuring impedance in 96 well plates, thus cells adhered to the bottom of the wells with micro-electrodes will increase electrical resistance which is recorded as a high cell index. Detachment of cells from the bottom of the plate will result lower electrical resistance, hence lower cell index values. The MTS assay targets the activity of mitochondrial activity of living cells.

Auranofin was used in this study as a positive control in both the MTS and xCELLigence assays. Originally, this drug was approved for the treatment of rheumatoid arthritis. Continued studies however have shown that auranofin (in its individual and combination treatments with other agents) exhibits anticancer activity by inhibiting thioredoxin reductase [49], and thus inducing apoptosis, among other anticancer mechanisms. A number of cancer cell lines that have shown susceptibility to auranofin include MCF-7 human breast cancer cells [50, 51], Hep3B human hepatocellular carcinoma cells [52], LNcap and 22RV1 human prostate cancer cells [53], SKOV3 ovarian cancer cells [54], HCT116 and HT-29 colorectal cancer cells [55], human glioblastoma multiforme cells [56], and 10 non-small lung cancer cell lines [57]. The cytotoxic mechanism of action of auranofin on UMG87 glioblastoma cells is still yet to be fully explained, however its xCELLigence profile in this study lead to the assumption that it has an intracellular target, most likely a gene involved in metabolism since low doses of the drug induced resistance and hypermetabolism (Fig. 3).

Considering the gap in knowledge about bioactive extracts from endophytic fungi, it became necessary to perform secondary metabolite profiling of the cytotoxic *Alternaria* sp. KTDL7’s crude extract. After analyzing LC-QTOF-MS/MS spectrum data for *Alternaria* sp. KDTL7’s crude extract for previously characterized compounds, seven secondary metabolites (Compounds **1** to **6**) were identified from compound libraries.

Compound **1** (1,8-dihydroxynaphthalene) is a key intermediate in the synthesis of dihydroxynaphtalene (DHN)-melanin, commonly found in fungi and is synthesized via the polyketide pathway [58]. Fungi within the *Alternaria* genus studied up to date have been shown to be DHN-melanin producers, including *A. alternata* 15A [59], and *A. infectoria* CBS 137.90 [60]. DHN-melanin was tested for antifungal activity on clinical isolates and was found to have a half-minimum inhibitory concentration (MIC_50_) of 128 μg/mL for *Aspergillus flavus*, 64 μg/mL for *A. niger*, 256 μg/mL for *A. fumigatus* and 512 μg/mL for *A. tamarii* [61].

Compound **2** (anserinone) is a polyketide that has been previously isolated from the cophrophilous *Podospora anserine*, where it was found to reduce radial growth of *Sordaria fimicola* and *Ascobolus furfuraceus* by 50 and 37% respectively [62, 63]. In that same study, anserinone B was found to be moderately cytotoxic with an average IC_50_ of 4.4 μg/mL after being tested on the National Cancer Institute’s 60 human tumor cell line panel [62, 64].

Compound **3** (phelligridin B) is a styrylpyrone derivative which is synthesized within the shikimate and acetate pathways [65]. This secondary metabolite has been found in ethanolic extracts of *Phellinus linteus* (Sang Huang) and has been shown to exhibit cytotoxic activity against Bel-7402 cells at an IC_50_ of 0.050 μM [66].

Compound **4** (metacytofilin) has been previously identified from the culture filtrate of *Metarhizium* sp. TA2759 and possess immunosuppressive properties [67]. It is a two-residue depsipeptide synthesized by non-ribosomal peptide synthases in combination with polyketide synthase [68].

Compound **5** (phomopsidin) is an interesting polyketide which has been previously isolated from a marine derived *Phomopsis* sp. TUF95F47 [69]. This secondary metabolite showed inhibition of microtubule assembly at an IC_50_ of 5.7 μM in the in vitro assembly analysis of porcine brain tubulin assay [70, 71].

Compound **6** (vermixocin) is a diphenyl ether derivative, previously isolated from a marine fungus, *Talaromyces* sp. LF458 [71]. Vermixocins were previously found to inhibit RNA synthesis as they interfered with incorporation of labeled uridine in a murine P388 leukemia cell line [72].

Commonly known secondary metabolites which have been previously identified in extracts of fungi from the *Alternaria* genus include alternariol, alternariol monomethyl ether, tentoxin, altesertin, alteichin, stemphyltoxin, altersolanol, altenusin and tenuazenoic acid were not detected in this study [73]. A possible explanation for this occurrence is that different fungal strains in the same genus have the biosynthetic capability of producing a wide variety of chemically diverse secondary metabolites [74]. The type of method and solvent used in the extraction process may also significantly affect the nature and quantity of secondary metabolites recovered [75]. Some researchers have reported the use of acidified extracting organic solvents or acidified filtrate broth to increase the solubility of fungal secondary metabolites in organic solvents [76].

## Conclusions

This study provides evidence that the ethyl acetate crude extract of *Alternaria* sp. KTDL7 exerts a notable dose-dependent and selective cytotoxic activity on UMG87 glioblastoma cells. Metabolite profiling also showed that *Alternaria s*p. KTDL7 is capable of producing compounds similar to those from terrestrial plants and marine fungi belonging to different genera, as is the case with compounds **2** to **6**. This further supports the notion that more complex chemical structural scaffolds with interesting bioactivities are likely to be harbored by fungal symbionts from diverse origins. Further studies will be aimed at isolating and characterizing the cytotoxic pure compounds from the crude extract of *Alternaria* sp. KTDL7 and determining their mechanism of action, which could result in the development of a fungal-based drug for glioblastoma multiforme treatment.

## Supplementary information

**Additional file 1.** Phylogenetic relationships of fungal endophytes from healthy leaves, stems and seeds of D. stramonium inferred based on ITS1 and ITS4 sequences. The numbers at branch nodes represent maximum likelihood bootstrap values from analyses with 1000 replicates. In boldface are fungal endophytes isolated from D. stramonium (GenBank accession number, name and isolate code). Fungal endophytes according to morphological characteristics on PDA plates, where group A are filamentous fungi, B and C being non-filamentous fungi. Evolutionary analyses were conducted in MEGA7 (Kumar et al., 2016).

**Additional file 2. **LC-QTOF-MS-MS_Analysis. Mass spectra for the crude extract of *Alternaria* sp. KTDL7 and the mass fragment patterns of the identified compounds: 1,8-dihydroxynaphthalene (**1**), anserinone B (**2**), phelligridin B (**3**), metacytofilin (**4**), phomopsidin (**5**) and vermixocin A (**6**).

## Data Availability

The datasets supporting the conclusions of this article are available in the Mendeley Data repository, https://data.mendeley.com/datasets/xt4br8zmtz/1

## Abbreviations

CID: Collision induced dissociation
DMSO: Dimethyl sulfoxide
ESI(+): Electrospray ionization (positive mode)
ITS: Internal transcribed spacer region
LC-QTOF-MS/MS: Liquid chromatography couple to quadrupole time-of-flight with tandem mass spectrometry
LSD: Least significant difference
MANOVA: Multivariate-analysis of variance
MTS: 3-(4,5-dimethylthiazol-2-yl)-5-(3-carboxymethoxyphenyl)-2-(4-sulfophenyl)-2*H*-tetrazolium
NCBI: National Center for Biotechnology Information
PBS: Phosphate buffered saline
PDA: Potato dextrose agar
PDB: Potato dextrose broth
PVDF: Polyvinylidene fluoride
rDNA: Ribosomal deoxyribonucleic acid
RP-UHPLC: Reverse phase ultra-high-performance liquid chromatography
RTCA: Real time cell analyzer
SD: Standard deviation
UHPLC: Ultra-high-performance liquid chromatography.
