# Supplementary material for: Cytotoxic activity of crude extracts from Datura stramonium’s fungal endophytes against A549 lung carcinoma and UMG87 glioblastoma cell lines and LC-QTOF-MS/MS based metabolite profiling
Source: BMC Complement Altern Med. 2019 Nov 21;19:330. doi: 10.1186/s12906-019-2752-9 (PMC6873518; doi:10.1186/s12906-019-2752-9)
Supplement: Supplementary file 1 — Additional file 1. Phylogenetic relationships of fungal endophytes from healthy leaves, stems and seeds of D. stramonium inferred based on ITS1 and ITS4 sequences. The numbers at branch nodes represent maximum likelihood bootstrap values from analyses with 1000 replicates. In boldface are fungal endophytes isolated from D. stramonium (GenBank accession number, name and isolate code). Fungal endophytes according to morphological characteristics on PDA plates, where group A are filamentous fungi, B and C being non-filamentous fungi. Evolutionary analyses were conducted in MEGA7 (Kumar et al., 2016). [file 12906_2019_2752_MOESM1_ESM.pdf]

SUPPLEMENTARY FILE 1

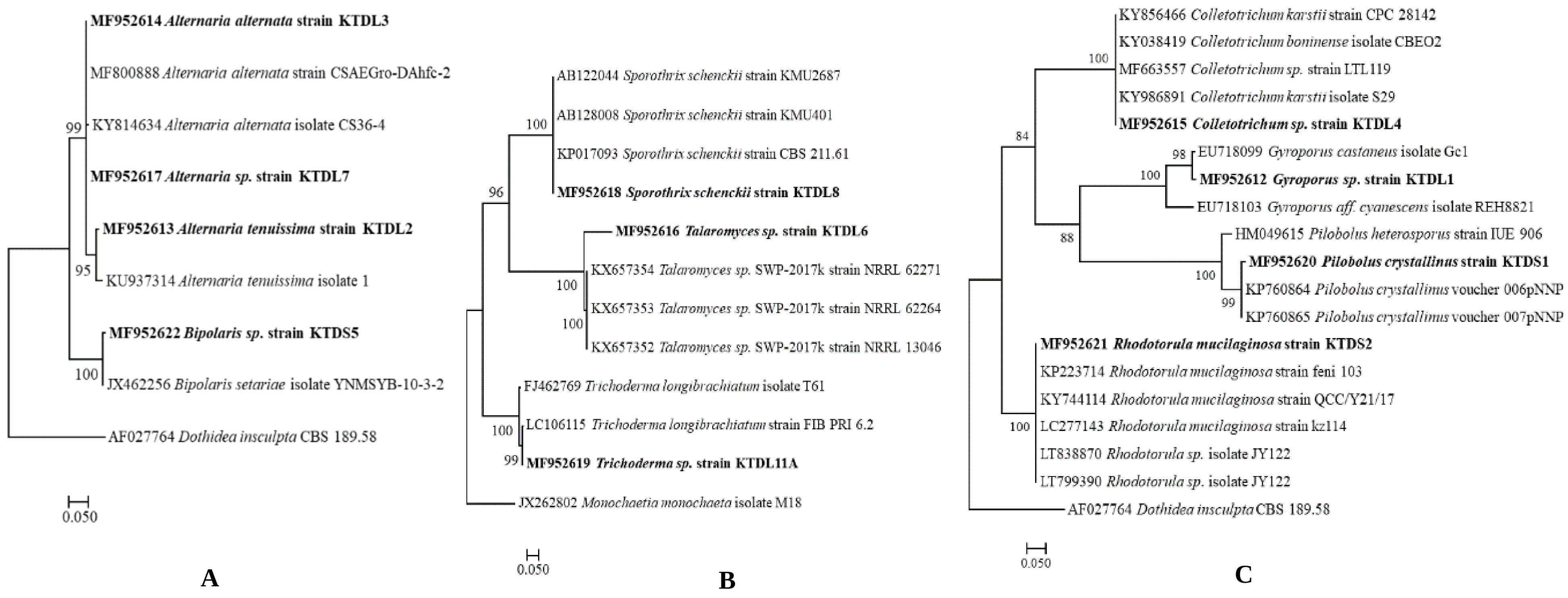

Figure S1-1: Phylogenetic relationships of fungal endophytes from healthy leaves, stems and seeds of *D. stramonium* inferred based on ITS1 and ITS4 sequences. The numbers at branch nodes represent maximum likelihood bootstrap values from analyses with 1000 replicates. In boldface are fungal endophytes isolated from *D. stramonium* (GenBank accession number, name and isolate code). Fungal endophytes according to morphological characteristics on PDA plates, where group **A** are filamentous fungi, **B** and **C** being non-filamentous fungi. Evolutionary analyses were conducted in MEGA7 (Kumar et al., 2016).
