# Supplementary material for: Cytotoxic activity of crude extracts from Datura stramonium’s fungal endophytes against A549 lung carcinoma and UMG87 glioblastoma cell lines and LC-QTOF-MS/MS based metabolite profiling
Source: BMC Complement Altern Med. 2019 Nov 21;19:330. doi: 10.1186/s12906-019-2752-9 (PMC6873518; doi:10.1186/s12906-019-2752-9)
Supplement: Supplementary file 2 — Additional file 2. LC-QTOF-MS-MS_Analysis. Mass spectra for the crude extract of Alternaria sp. KTDL7 and the mass fragment patterns of the identified compounds: 1,8-dihydroxynaphthalene (1), anserinone B (2), phelligridin B (3), metacytofilin (4), phomopsidin (5) and vermixocin A (6). [file 12906_2019_2752_MOESM2_ESM.pdf]

SUPPLEMENTARY FILE 2

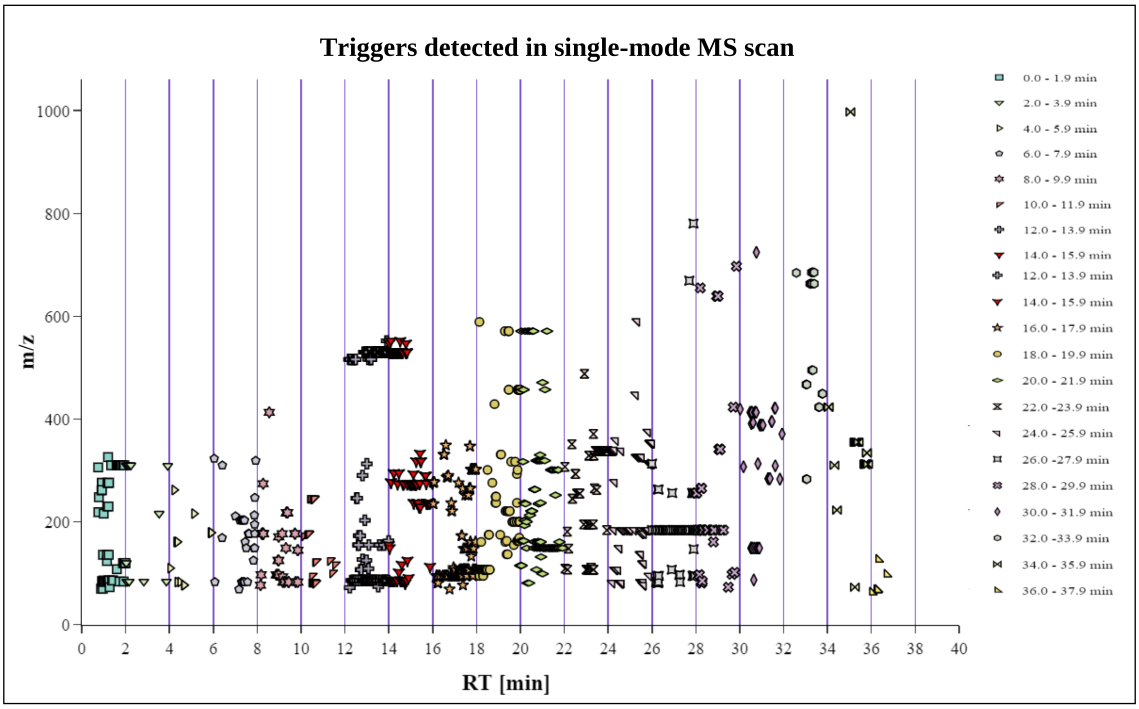

Figure S2-1: Scatter plots showing triggers detected in single mode-MS ESI(+). Each dot represents a molecular ion with *m/z* value plotted against retention time (RT). Number of analytes eluted with higher molecular mass was directly proportional to RT, suggesting that these were non-polar compounds.

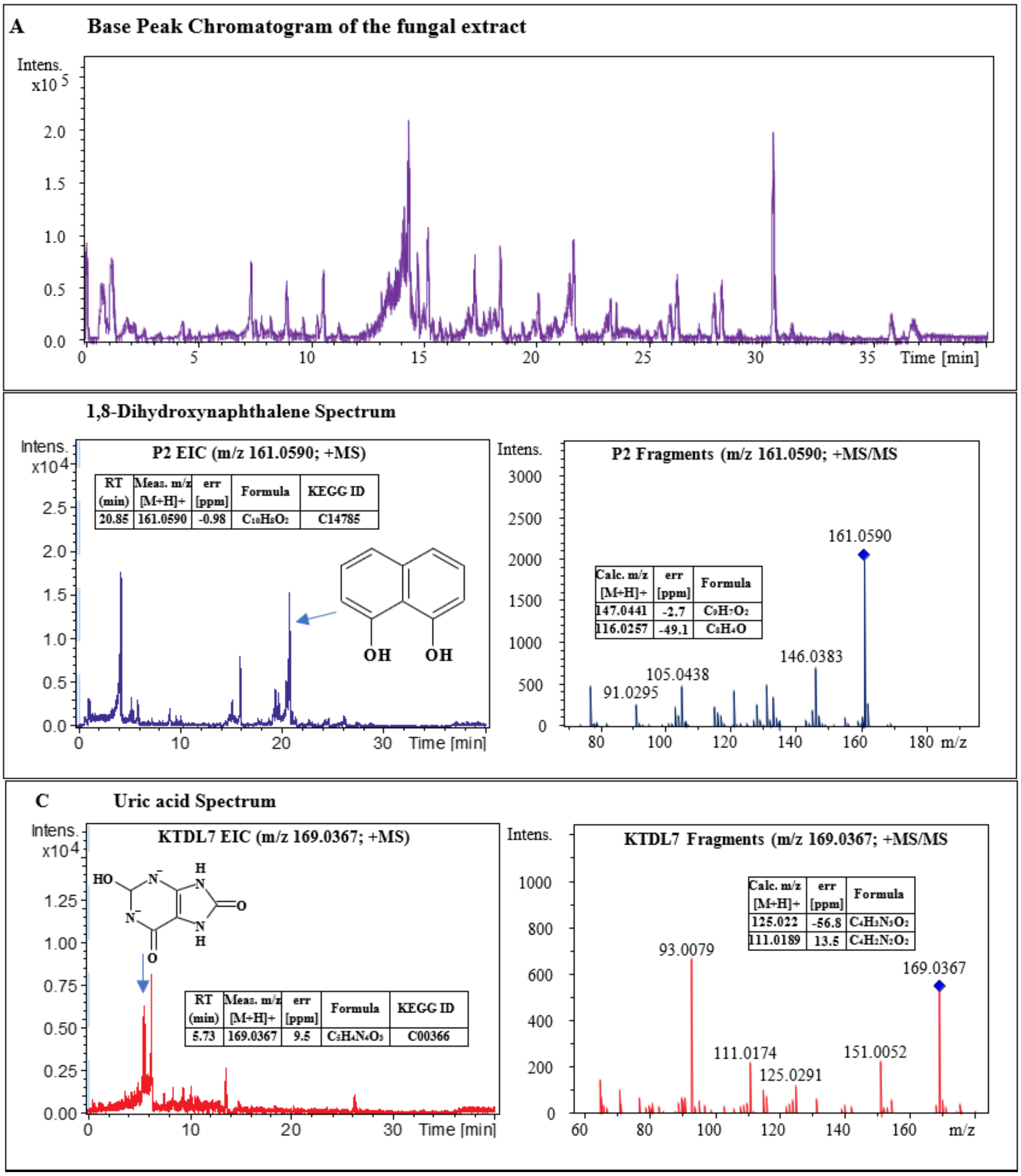

Figure S2-2: **(A)**The base peak chromatogram resulting the fungal extract. **(B)** and **(C)** show the extracted ion chromatogram (EIC) of 1,8-Dihydroxynaphthalene and uric acid (left) respectively with their CID fragments (right). **Meas. *m/z*** denotes measured *m/z*, while **Calc. *m/z*** denotes calculated *m/z*. Ion mass of all fragment species in this figure was [M+H]+.

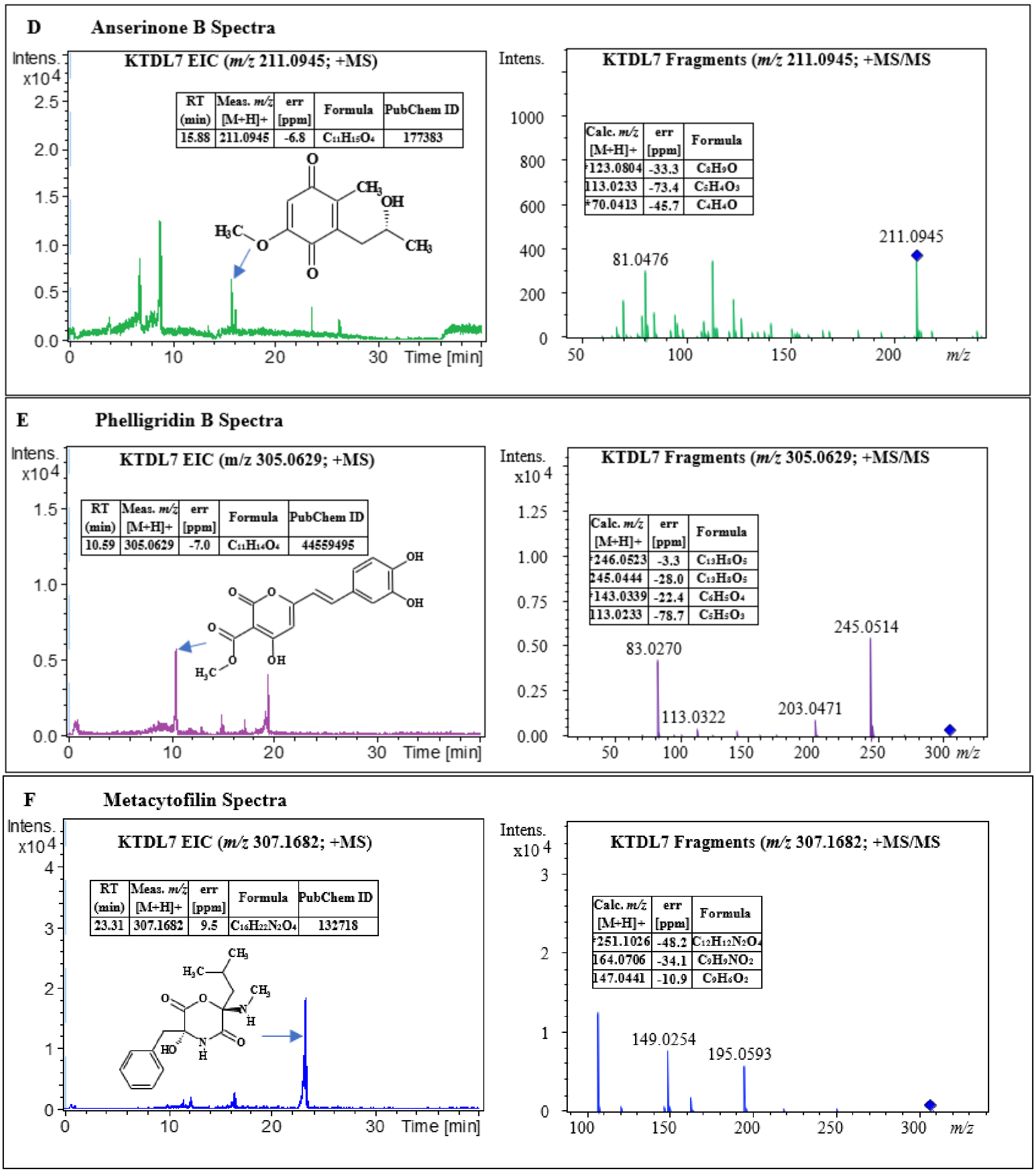

Figure S2-3: Extracted ion chromatogram (EIC) of **(D)** Anserinone B, **(E)** Phelligridin B and **(F)** Metacytofilin on the left and the CID fragments on the right. **Meas. *m/z*** denotes measured *m/z*, while * denotes fragments with a calculated *m/z* (**Calc. *m/z***) of [M+2H]+.

Alternaria sp. KTDL7 LC-QTOF-MS Spectrum data

#	*m/z*	Res.	S/N	I	I %	FWHM
1	69.0243	8250	5.3	146	1.2	0.0084
2	85.0057	9535	3.8	108	0.9	0.0089
3	86.0737	9098	8.2	230	1.9	0.0095
4	89.036	9432	3.6	101	0.8	0.0094
5	90.9528	9016	35.6	1012	8.3	0.0101
6	99.0183	9640	5.6	160	1.3	0.0103
7	100.0499	9709	5.2	151	1.2	0.0103
8	100.9975	9537	8.9	257	2.1	0.0106
9	105.043	9989	3.6	105	0.9	0.0105
10	107.0219	9643	40.9	1200	9.8	0.0111
11	108.0236	8599	3.5	103	0.8	0.0126
12	109.0736	10427	4.2	124	1	0.0105
13	111.0888	10150	5.6	165	1.4	0.0109
14	115.0104	9797	6.4	192	1.6	0.0117
15	115.083	10096	3.8	114	0.9	0.0114
16	116.0422	10344	8	238	1.9	0.0112
17	116.0785	9796	3.6	108	0.9	0.0118
18	118.0574	10082	11.1	332	2.7	0.0117
19	121.0351	10363	6.9	206	1.7	0.0117
20	123.0867	10301	5.4	163	1.3	0.0119
21	124.0094	10161	4	120	1	0.0122
22	127.0085	10335	13.8	418	3.4	0.0123
23	129.0217	10480	18	547	4.5	0.0123
24	130.0199	9298	12.3	375	3.1	0.014
25	130.1276	10554	4.5	136	1.1	0.0123
26	132.0707	10113	15.2	466	3.8	0.0131

#	*m/z*	Res.	S/N	I	I %	FWHM
27	135.0701	10118	5.4	168	1.4	0.0133
28	136.0319	7692	3.8	117	1	0.0177
29	139.0789	10631	3.8	118	1	0.0131
30	141.9252	10671	7	218	1.8	0.0133
31	144.0323	10584	8.9	279	2.3	0.0136
32	145.0158	10626	21.3	668	5.5	0.0136
33	148.9887	10375	36.9	1165	9.5	0.0144
34	149.0247	10567	16.1	507	4.1	0.0141
35	149.9919	11108	3.3	103	0.8	0.0135
36	151.0759	10539	4.5	144	1.2	0.0143
37	155.071	10666	10.4	332	2.7	0.0145
38	157.0489	10219	3.7	117	1	0.0154
39	157.0861	10622	3.5	112	0.9	0.0148
40	158.1183	11020	7	226	1.8	0.0143
41	158.9284	10750	20.7	663	5.4	0.0148
42	161.0494	5632	8.3	267	2.2	0.0286
43	163.0239	9821	5.3	172	1.4	0.0166
44	163.0782	8507	3.7	120	1	0.0192
45	166.0505	9330	6.3	204	1.7	0.0178
46	167.0328	10898	15.3	497	4.1	0.0153
47	171.0632	10726	20.3	663	5.4	0.0159
48	173.0787	10764	5.2	172	1.4	0.0161
49	177.0158	11360	5.6	186	1.5	0.0156
50	177.0511	10461	3.3	108	0.9	0.0169
51	180.0497	6882	3.3	108	0.9	0.0262
52	185.0768	8075	3.3	111	0.9	0.0229
53	189.0713	10367	12.1	406	3.3	0.0182
54	191.1223	10958	5.9	199	1.6	0.0174
55	195.0595	10984	45.3	1527	12.5	0.0178
56	196.0622	10750	5.9	200	1.6	0.0182
57	197.0776	8770	12.1	409	3.3	0.0225

#	*m/z*	Res.	S/N	I	I %	FWHM
58	198.062	6967	4.3	145	1.2	0.0284
59	199.1261	11467	4.5	151	1.2	0.0174
60	200.1574	11174	5.1	173	1.4	0.0179
61	203.0103	11137	3.8	130	1.1	0.0182
62	203.0489	10406	29.9	1021	8.4	0.0195
63	204.0509	9387	3.6	124	1	0.0217
64	205.042	11005	47.3	1621	13.3	0.0186
65	206.0452	11094	6.3	216	1.8	0.0186
66	206.1062	7970	5.5	189	1.5	0.0259
67	207.1186	6388	3.9	135	1.1	0.0324
68	211.05	9538	4.6	160	1.3	0.0221
69	211.0981	10871	16.1	556	4.5	0.0194
70	213.0682	10068	4.9	170	1.4	0.0212
71	213.1021	10001	5.9	206	1.7	0.0213
72	215.0828	9801	3.5	123	1	0.0219
73	218.9816	11093	10.9	382	3.1	0.0197
74	221.0682	9815	8	281	2.3	0.0225
75	221.1036	10953	3	104	0.8	0.0202
76	223.0521	9424	3	106	0.9	0.0237
77	223.0854	10972	7.2	252	2.1	0.0203
78	224.0374	11047	6.4	224	1.8	0.0203
79	226.9053	11092	65.2	2297	18.8	0.0205
80	227.0915	9372	7.1	249	2	0.0242
81	229.0948	9334	4	141	1.2	0.0245
82	231.0752	10332	4	141	1.2	0.0224
83	239.1126	11135	3.8	135	1.1	0.0215
84	239.1876	11702	9.2	325	2.7	0.0204
85	245.052	11264	32.8	1169	9.6	0.0218
86	246.0558	10933	4.1	148	1.2	0.0225
87	251.1144	9906	9.4	336	2.7	0.0254
88	255.18	11876	4	143	1.2	0.0215

#	*m/z*	Res.	S/N	I	I %	FWHM
89	257.1954	11897	13.8	492	4	0.0216
90	261.0739	8424	5.4	194	1.6	0.031
91	262.0796	8293	2.8	101	0.8	0.0316
92	263.0599	11114	9.6	343	2.8	0.0237
93	263.184	11568	5.7	204	1.7	0.0228
94	265.1261	11286	6.9	245	2	0.0235
95	265.1987	11860	2.8	100	0.8	0.0224
96	266.0713	11218	9.8	348	2.8	0.0237
97	269.1931	12428	4.1	146	1.2	0.0217
98	274.2201	11490	60.4	2136	17.5	0.0239
99	275.2216	11004	11.1	394	3.2	0.025
100	276.0908	11425	8	282	2.3	0.0242
101	277.1237	11599	8.5	301	2.5	0.0239
102	279.1036	11512	54.4	1926	15.8	0.0242
103	279.1742	10872	4.8	171	1.4	0.0257
104	280.0963	5481	10.8	380	3.1	0.0511
105	281.1921	11440	15.1	531	4.3	0.0246
106	282.1966	8201	3	105	0.9	0.0344
107	283.2069	11290	10.9	384	3.1	0.0251
108	284.0855	10797	3	107	0.9	0.0263
109	285.0384	11863	3.9	138	1.1	0.024
110	285.1497	10629	4.7	165	1.4	0.0268
111	291.1365	10976	3.7	128	1	0.0265
112	293.1188	10990	8.2	284	2.3	0.0267
113	294.0994	10740	30	1043	8.5	0.0274
114	294.8821	12225	3.1	108	0.9	0.0241
115	295.1082	8190	3.9	136	1.1	0.036
116	295.1659	10905	8	280	2.3	0.0271
117	296.1636	11402	29.8	1034	8.5	0.026
118	297.1709	8287	6.4	221	1.8	0.0359
119	301.0835	11428	16.9	585	4.8	0.0263

#	*m/z*	Res.	S/N	I	I %	FWHM
120	302.0868	11169	3.1	106	0.9	0.027
121	302.2456	11770	5.5	190	1.6	0.0257
122	305.0623	9081	2.9	101	0.8	0.0336
123	307.1662	10188	4.1	141	1.2	0.0301
124	309.1471	9587	7.1	242	2	0.0322
125	311.127	11260	12.6	428	3.5	0.0276
126	312.0626	12027	7.9	269	2.2	0.0259
127	312.2658	11824	4.8	164	1.3	0.0264
128	313.1776	11886	13.9	472	3.9	0.0263
129	314.0777	11887	7.7	261	2.1	0.0264
130	318.239	12094	7.1	239	2	0.0263
131	322.0878	12146	9.9	328	2.7	0.0265
132	324.1908	11640	10.9	361	3	0.0279
133	325.1393	11195	8.2	269	2.2	0.029
134	327.0427	11874	4.1	134	1.1	0.0275
135	328.0768	12065	12.6	411	3.4	0.0272
136	328.15	11626	6	195	1.6	0.0282
137	329.1521	7696	5.1	168	1.4	0.0428
138	330.072	10989	3.1	101	0.8	0.03
139	331.2199	11591	6.3	203	1.7	0.0286
140	333.1045	11313	3.2	104	0.9	0.0294
141	338.2773	12622	10.3	330	2.7	0.0268
142	339.155	8744	3.6	115	0.9	0.0388
143	342.0755	11859	5.8	181	1.5	0.0288
144	347.1182	10861	3.7	115	0.9	0.032
145	362.8597	12536	18.4	542	4.4	0.0289
146	391.2123	12134	38	1004	8.2	0.0322
147	392.2152	12536	10.4	274	2.2	0.0313
148	396.981	12705	4.8	125	1	0.0312
149	403.1589	12323	7.7	194	1.6	0.0327
150	408.2361	12332	10.7	266	2.2	0.0331

#	*m/z*	Res.	S/N	I	I %	FWHM
151	413.1908	12402	9.8	240	2	0.0333
152	425.1378	11206	4.9	115	0.9	0.0379
153	430.8373	12593	30.5	705	5.8	0.0342
154	435.2158	11951	6.6	150	1.2	0.0364
155	452.2401	12256	13.7	296	2.4	0.0369
156	457.1955	11795	10.3	220	1.8	0.0388
157	498.815	13079	21.5	405	3.3	0.0381
158	516.1413	12781	19.8	357	2.9	0.0404
159	517.144	12385	5.6	100	0.8	0.0418
160	530.1566	12229	699.2	12223	100	0.0434
161	531.1593	12395	202.8	3539	29	0.0429
162	532.1554	12378	93.2	1620	13.3	0.043
163	533.1595	12128	28.4	493	4	0.044
164	534.1601	10776	6.8	118	1	0.0496
165	540.2846	12036	7.4	126	1	0.0449
166	547.1804	12589	189.9	3172	25.9	0.0435
167	548.1826	12986	54.6	912	7.5	0.0422
168	549.1789	11794	24	399	3.3	0.0466
169	552.1349	12786	41.8	690	5.6	0.0432
170	553.1374	12759	11.9	197	1.6	0.0434
171	561.3911	12647	6.2	100	0.8	0.0444
172	566.7932	13171	24.3	390	3.2	0.043
173	571.0613	12651	8.1	129	1.1	0.0451
174	579.1927	12899	21.7	341	2.8	0.0449
175	580.1956	12473	7.5	118	1	0.0465
176	634.7713	13501	28.9	419	3.4	0.047
177	668.7603	14537	12	169	1.4	0.046
178	702.7496	13490	35.5	483	4	0.0521
179	736.7385	13998	13.6	177	1.5	0.0526
180	744.2473	12608	8.4	108	0.9	0.059
181	758.4449	13179	52.8	671	5.5	0.0576

#	*m/z*	Res.	S/N	I	I %	FWHM
182	759.448	13057	23.1	293	2.4	0.0582
183	770.728	13742	28.5	359	2.9	0.0561
184	784.4551	12907	16.9	211	1.7	0.0608
185	798.4593	12890	33.5	410	3.4	0.0619
186	799.4621	13061	17.8	217	1.8	0.0612
187	803.4141	12380	250.9	3052	25	0.0649
188	804.4173	12187	133.1	1618	13.2	0.066
189	804.7169	13751	13.1	159	1.3	0.0585
190	805.4201	12353	38.9	473	3.9	0.0652
191	806.426	12612	9.9	120	1	0.0639
192	838.7063	13622	33.4	389	3.2	0.0616
193	842.3398	12078	12.7	148	1.2	0.0697
194	860.3504	12854	14.9	169	1.4	0.0669
195	861.3585	13354	12.7	144	1.2	0.0645
196	872.695	14770	12.7	141	1.2	0.0591
197	877.3747	12459	9.8	109	0.9	0.0704
198	906.6845	14628	24.8	267	2.2	0.062
199	940.6728	14644	11.8	123	1	0.0642
200	974.6626	14881	19.7	199	1.6	0.0655
201	1042.6408	14838	12.8	124	1	0.0703
202	1059.3272	12746	459.7	4474	36.6	0.0831
203	1060.3297	12850	267.2	2601	21.3	0.0825
204	1061.3277	12874	160.2	1562	12.8	0.0824
205	1062.3281	12948	62.5	611	5	0.082
206	1063.3271	13605	21.6	212	1.7	0.0782
207	1076.3504	13846	120.7	1194	9.8	0.0777
208	1077.353	13638	66.6	660	5.4	0.079
209	1078.3499	13523	39.4	391	3.2	0.0797
210	1079.3512	12710	14	139	1.1	0.0849
211	1081.3065	13029	19.2	192	1.6	0.083
212	1082.3076	13060	10.4	104	0.9	0.0829

#	*m/z*	Res.	S/N	I	I %	FWHM
213	1193.6423	13708	22.4	247	2	0.0871
214	1194.6447	13691	17.2	190	1.6	0.0873

APPENDIX V

Compound molecular features (Auto MSn)

#	RT [min]	Area	Int. Type	I	S/N	Chromatogram	Max. m/z	FWHM [min]	Group
1	0.11	5266219.5	MS(n)	1217601	366.4	MS(n): TIC +All MS	226.9515	0.07	0.0 - 1.9 min
2	0.12	89574.5	MolFeature	15164	39.9		413.2654		0.0 - 1.9 min
3	0.13	68790.6	MolFeature	5807	64.5		873.3337		0.0 - 1.9 min
4	0.13	34540.1	MolFeature	3363	37.4		635.8798		0.0 - 1.9 min
5	0.13	21812.2	MolFeature	2077	23.1		431.9171		0.0 - 1.9 min
6	0.13	62902.9	MolFeature	5224	58		941.3205		0.0 - 1.9 min
7	0.13	42776.9	MolFeature	4625	51.4		703.8661		0.0 - 1.9 min
8	0.14	32093.7	MolFeature	5683	12.7		158.964		0.0 - 1.9 min
9	0.71	30028.1	MolFeature	1686	3.9		274.1397		0.0 - 1.9 min
10	0.72	49391.6	MolFeature	3259	5.8		413.2127		0.0 - 1.9 min
11	0.74	198406	Chromatogram	54142	70	BPC +All MS	219.0266	0.06	0.0 - 1.9 min
12	0.75	67310.7	MolFeature	5383	6		251.1602		0.0 - 1.9 min
13	0.75	50035.5	MolFeature	4189	20.4		504.1915		0.0 - 1.9 min
14	0.75	5474755	MS(n)	582320	174.3	MS(n): TIC +All MS	342.1395	0.12	0.0 - 1.9 min
15	0.75	5474755	MS(n)	582320	174.3	MS(n): TIC +All MS	342.1395	0.12	0.0 - 1.9 min
16	0.75	18396.9	MolFeature	2193	8.3		543.133		0.0 - 1.9 min
17	0.76	328156.1	MolFeature	31773	118.2		342.1396		0.0 - 1.9 min
18	0.76	53688.3	MolFeature	7381	3.8		723.1962		0.0 - 1.9 min
19	0.76	176656.6	MolFeature	26543	56		381.0797		0.0 - 1.9 min
20	0.77	35775.7	MolFeature	3949	8.1		180.0866		0.0 - 1.9 min
21	0.77	10467.6	MolFeature	927	3.3		667.2286		0.0 - 1.9 min
22	0.77	17920.6	MolFeature	1865	6.6		522.2036		0.0 - 1.9 min
23	0.77	20491.8	MolFeature	1903	6.5		428.1767		0.0 - 1.9 min
24	0.77	29544.3	MolFeature	2766	5.6		261.0421		0.0 - 1.9 min
25	0.78	5962.8	MolFeature	426	9.5		1153.3894		0.0 - 1.9 min
26	0.78	6050.2	MolFeature	510	5.7		685.2431		0.0 - 1.9 min
27	0.78	9934.4	MolFeature	915	3.6		476.1611		0.0 - 1.9 min
28	0.78	56698.1	MolFeature	7518	24.1		360.15		0.0 - 1.9 min
29	0.78	8808.5	MolFeature	978	3.4		252.1077		0.0 - 1.9 min
30	0.79	20876.2	MolFeature	1237	4.6		395.2008		0.0 - 1.9 min
31	0.79	7674.1	MolFeature	503	5.6		829.2839		0.0 - 1.9 min
32	0.79	17871.1	MolFeature	1443	6.2		487.1692		0.0 - 1.9 min
33	0.79	24317.7	MolFeature	2578	4.1		344.1369		0.0 - 1.9 min
34	0.79	59751.6	MolFeature	8659	9.8		118.0865		0.0 - 1.9 min
35	0.79	14295.6	MolFeature	1335	5.6		381.1858		0.0 - 1.9 min
36	0.79	9313.1	MolFeature	939	3.5		423.1971		0.0 - 1.9 min
37	0.80	12368.5	MolFeature	1129	6.8		409.1819		0.0 - 1.9 min
38	0.80	264364.8	MolFeature	26144	106		266.1237		0.0 - 1.9 min
39	0.80	19289.9	MolFeature	2194	6.5		248.1132		0.0 - 1.9 min

#	RT [min]	Area	Int. Type	I	S/N	Chromatogram	Max. m/z	FWHM [min]	Group
40	0.80	10037.4	MolFeature	1131	4.5		292.1033		0.0 - 1.9 min
41	0.80	49265.1	MolFeature	3100	3.4		707.2211		0.0 - 1.9 min
42	0.80	18400.2	MolFeature	2138	11.3		351.176		0.0 - 1.9 min
43	0.80	214081.8	MolFeature	16102	50.1		325.1132		0.0 - 1.9 min
44	0.80	24695	MolFeature	2528	3.1		262.1287		0.0 - 1.9 min
45	0.80	12995.5	MolFeature	1537	7.7		278.1234		0.0 - 1.9 min
46	0.80	16652.5	MolFeature	2048	5.9		276.1445		0.0 - 1.9 min
47	0.80	53191.7	MolFeature	7204	13.3		294.1551		0.0 - 1.9 min
48	0.80	16621.5	MolFeature	1845	8		116.0709		0.0 - 1.9 min
49	0.80	19479.4	MolFeature	1869	3.1		145.0499		0.0 - 1.9 min
50	0.81	37116.1	MolFeature	4217	6.6		130.0502		0.0 - 1.9 min
51	0.81	116728.3	MolFeature	9918	7.3		163.0604		0.0 - 1.9 min
52	0.81	70524	MolFeature	7557	6.7		280.139		0.0 - 1.9 min
53	0.81	12097.9	MolFeature	1249	5.4		310.1171		0.0 - 1.9 min
54	0.82	16991	MolFeature	2281	25.3		328.1377		0.0 - 1.9 min
55	0.83	17665.3	MolFeature	2519	5.6		393.223		0.0 - 1.9 min
56	0.86	149199.2	MolFeature	7289	4.8		365.1053		0.0 - 1.9 min
57	0.92	407503.3	Chromatogram	45166	58.3	BPC +All MS	203.0528	0.14	0.0 - 1.9 min
58	0.93	606165.9	MolFeature	45531	147		203.0528		0.0 - 1.9 min
59	0.93	345512.5	MolFeature	25225	4.7		383.1161		0.0 - 1.9 min
60	0.94	15205.5	MolFeature	2571	4.1		140.0685		0.0 - 1.9 min
61	0.95	17910.4	MolFeature	1279	3.2		217.0681		0.0 - 1.9 min
62	0.96	75064.9	MolFeature	5972	7.6		233.0633		0.0 - 1.9 min
63	1.00	93047.1	MolFeature	6030	25.1		245.0646		0.0 - 1.9 min
64	1.07	50490.3	MolFeature	9734	8.7		280.1395		0.0 - 1.9 min
65	1.07	16224.9	MolFeature	2987	3.7		262.1281		0.0 - 1.9 min
66	1.08	784494.8	MS(n)	113585	32.8	MS(n): TIC +All MS	294.1551	0.07	0.0 - 1.9 min
67	1.08	784494.8	MS(n)	113585	32.8	MS(n): TIC +All MS	294.1551	0.07	0.0 - 1.9 min
68	1.09	45604.8	MolFeature	11217	17.5		130.0502		0.0 - 1.9 min
69	1.11	11036.8	MolFeature	827	3.5		381.1876		0.0 - 1.9 min
70	1.13	126401.9	Chromatogram	14219	16.3	BPC +All MS	294.1547	0.2	0.0 - 1.9 min
71	1.14	28568.9	MolFeature	2007	3.2		344.1339		0.0 - 1.9 min
72	1.18	179799	MolFeature	13344	24.5		294.1548		0.0 - 1.9 min
73	1.19	53827	MolFeature	3506	10.1		276.1443		0.0 - 1.9 min
74	1.21	21467.4	MolFeature	1917	3.9		132.1018		0.0 - 1.9 min
75	1.22	11701.2	MolFeature	800	5.5		456.2074		0.0 - 1.9 min
76	1.53	9908.6	MolFeature	579	3.7		365.1914		0.0 - 1.9 min
77	1.75	101223.4	MolFeature	6094	14		328.1391		0.0 - 1.9 min
78	1.75	22495.9	MolFeature	1250	4.7		310.1286		0.0 - 1.9 min
79	2.43	30262.9	MolFeature	1413	4.8		393.2225		2.0 - 3.9 min
80	2.73	66114.5	MolFeature	4913	32.8		243.0835		2.0 - 3.9 min
81	2.94	28028.9	MolFeature	1026	3.4		391.2059		2.0 - 3.9 min
82	3.00	33848	MolFeature	1352	5.3		407.2384		2.0 - 3.9 min

#	RT [min]	Area	Int. Type	I	S/N	Chromatogram	Max. m/z	FWHM [min]	Group
83	4.66	14345	MolFeature	1785	5.5		253.1041		4.0 - 5.9 min
84	4.70	227738.5	MolFeature	16782	81.6		285.0942		4.0 - 5.9 min
85	4.70	51770	MolFeature	3949	17		203.0912		4.0 - 5.9 min
86	4.70	56001.7	MolFeature	4277	4.8		547.1982		4.0 - 5.9 min
87	4.71	40488.9	MolFeature	2808	7.8		263.1124		4.0 - 5.9 min
88	4.71	32463.3	MolFeature	2408	6.1		245.1016		4.0 - 5.9 min
89	4.72	184742	Chromatogram	23581	31.2	BPC +All MS	285.0942	0.09	4.0 - 5.9 min
90	4.73	688372.7	MS(n)	71135	20.5	MS(n): TIC +All MS	285.0942	0.18	4.0 - 5.9 min
91	4.73	688372.7	MS(n)	71135	20.5	MS(n): TIC +All MS	285.0942	0.18	4.0 - 5.9 min
92	4.74	7871.1	MolFeature	636	3.4		330.1329		4.0 - 5.9 min
93	4.74	42143.6	MolFeature	2468	6.8		312.1224		4.0 - 5.9 min
94	4.75	23199.5	MolFeature	1155	3.3		614.2195		4.0 - 5.9 min
95	4.75	21786.6	MolFeature	1512	3.1		352.1144		4.0 - 5.9 min
96	4.93	10168.5	MolFeature	1393	3.7		213.1119		4.0 - 5.9 min
97	4.93	46520.9	MolFeature	6977	3.3		253.1047		4.0 - 5.9 min
98	4.94	12118.2	MolFeature	1697	18.9		231.1227		4.0 - 5.9 min
99	4.94	31741.8	Chromatogram	8494	10.6	BPC +All MS	253.1046	0.07	4.0 - 5.9 min
100	5.16	4018.9	MolFeature	410	4.6		588.4076		4.0 - 5.9 min
101	5.18	62564.1	MolFeature	4128	5.8		312.1224		4.0 - 5.9 min
102	5.18	87427.6	MolFeature	4915	8.6		681.2399		4.0 - 5.9 min
103	5.19	26252	MolFeature	2174	4.2		330.1333		4.0 - 5.9 min
104	5.78	205483.4	MS(n)	44212	12.2	MS(n): TIC +All MS	211.0939	0.07	4.0 - 5.9 min
105	5.78	205483.4	MS(n)	44212	12.2	MS(n): TIC +All MS	211.0939	0.07	4.0 - 5.9 min
106	5.78	52512	Chromatogram	12351	16.1	BPC +All MS	211.0939	0.07	4.0 - 5.9 min
107	5.78	65689.9	MolFeature	11822	7		211.0939		4.0 - 5.9 min
108	6.29	3496.3	MolFeature	446	5		689.4923		6.0 - 7.9 min
109	6.32	30800.6	MolFeature	2865	9.2		314.1383		6.0 - 7.9 min
110	6.32	19782.1	MolFeature	1694	4.2		336.1203		6.0 - 7.9 min
111	6.75	16753.3	MolFeature	2002	5		336.1204		6.0 - 7.9 min
112	6.75	42306.3	MolFeature	5068	8.4		314.1385		6.0 - 7.9 min
113	6.92	10671.3	MolFeature	1682	18.7		288.1225		6.0 - 7.9 min
114	6.92	19373.7	MolFeature	2474	6.6		310.1051		6.0 - 7.9 min
115	6.96	18578.6	MolFeature	1947	5.6		852.5088		6.0 - 7.9 min
116	7.10	32220.8	MolFeature	4402	5.2		706.4499		6.0 - 7.9 min
117	7.10	38534.8	MolFeature	5462	14.7		245.1019		6.0 - 7.9 min
118	7.10	104831.9	MolFeature	11976	78.5		327.1049		6.0 - 7.9 min
119	7.11	40275.4	MolFeature	4430	8.8		322.1493		6.0 - 7.9 min
120	7.12	84462.8	Chromatogram	12921	17	BPC +All MS	327.1049	0.11	6.0 - 7.9 min
121	7.12	380594.6	MS(n)	45705	12.3	MS(n): TIC +All MS	327.1048	0.11	6.0 - 7.9 min
122	7.48	6292	MolFeature	579	6.4		411.1062		6.0 - 7.9 min
123	7.50	4644.6	MolFeature	564	3.7		647.234		6.0 - 7.9 min
124	7.50	13253.8	MolFeature	1324	4.6		625.2521		6.0 - 7.9 min

#	RT [min]	Area	Int. Type	I	S/N	Chromatogram	Max. m/z	FWHM [min]	Group
125	7.51	6838.7	MolFeature	372	4		663.227		6.0 - 7.9 min
126	7.83	23502.7	MolFeature	2056	10.3		442.337		6.0 - 7.9 min
127	7.84	20300	MolFeature	1114	3.8		425.3099		6.0 - 7.9 min
128	7.85	14360.9	MolFeature	1192	4.2		447.2927		6.0 - 7.9 min
129	7.92	7155.6	MolFeature	1387	7.8		279.1561		6.0 - 7.9 min
130	8.18	6681.3	MolFeature	282	3.1		647.2357		8.0 - 9.9 min
131	8.44	399519.6	MS(n)	93779	26.6	MS(n): TIC +All MS	353.2291	0.06	8.0 - 9.9 min
132	8.44	15851.9	MolFeature	2551	9.7		427.2646		8.0 - 9.9 min
133	8.44	52222.9	Chromatogram	14260	19	BPC +All MS	353.2292	0.06	8.0 - 9.9 min
134	8.45	23783.9	MolFeature	4973	3.5		295.2261		8.0 - 9.9 min
135	8.45	66986.2	MolFeature	9468	105.2		683.4684		8.0 - 9.9 min
136	8.45	69324.1	MolFeature	12569	12.5		353.2292		8.0 - 9.9 min
137	8.45	34436.9	MolFeature	6152	6.3		348.2738		8.0 - 9.9 min
138	8.46	18775	MolFeature	2952	8.5		173.1171		8.0 - 9.9 min
139	8.48	4941.9	MolFeature	950	4.3		293.2105		8.0 - 9.9 min
140	8.49	13766.1	MolFeature	2972	23.8		351.2131		8.0 - 9.9 min
141	8.56	25022.9	MolFeature	2172	4.8		647.2358		8.0 - 9.9 min
142	8.59	29599	MolFeature	4580	50.9		568.2957		8.0 - 9.9 min
143	8.59	34399.2	MolFeature	4838	53.8		573.2511		8.0 - 9.9 min
144	8.63	17361.5	MolFeature	3191	3		246.2427		8.0 - 9.9 min
145	8.74	8484.2	MolFeature	1294	4.9		538.2103		8.0 - 9.9 min
146	8.77	35813.8	MolFeature	2364	9.9		500.3782		8.0 - 9.9 min
147	8.77	17746	MolFeature	1423	5.3		483.3519		8.0 - 9.9 min
148	8.78	19897.4	MolFeature	1252	7.4		505.3339		8.0 - 9.9 min
149	8.99	47529.2	MolFeature	5568	61.9		820.3954		8.0 - 9.9 min
150	8.99	1323402.3	MS(n)	322275	94.1	MS(n): TIC +All MS	516.2306	0.06	8.0 - 9.9 min
151	8.99	252752.8	Chromatogram	64781	86.7	BPC +All MS	516.2306	0.06	8.0 - 9.9 min
152	9.00	329943	MolFeature	60903	203.3		516.2306		8.0 - 9.9 min
153	9.00	31119.7	MolFeature	3593	9.4		540.2108		8.0 - 9.9 min
154	9.00	196732.3	MolFeature	34618	98.1		533.2569		8.0 - 9.9 min
155	9.00	27367.2	MolFeature	4090	3.3		535.2556		8.0 - 9.9 min
156	9.00	191202	MolFeature	29224	111.7		538.2119		8.0 - 9.9 min
157	9.00	128523	MolFeature	12534	139.3		1053.4367		8.0 - 9.9 min
158	9.00	40895.2	MolFeature	6730	3.5		518.2297		8.0 - 9.9 min
159	9.00	8044.5	MolFeature	786	3.9		554.1855		8.0 - 9.9 min
160	9.12	18036.5	MolFeature	1533	3.1		625.253		8.0 - 9.9 min
161	9.21	87653	Chromatogram	18524	24.1	BPC +All MS	353.2297	0.06	8.0 - 9.9 min
162	9.21	774773.9	MS(n)	115464	31.5	MS(n): TIC +All MS	353.2296	0.06	8.0 - 9.9 min
163	9.22	31649.1	MolFeature	5304	16.9		313.2368		8.0 - 9.9 min
164	9.22	6091.4	MolFeature	606	6.7		705.4481		8.0 - 9.9 min
165	9.22	37366.3	MolFeature	6753	15.6		173.1173		8.0 - 9.9 min
166	9.23	92052.4	MolFeature	11836	10.3		683.4691		8.0 - 9.9 min
167	9.23	30695.5	MolFeature	4591	30		295.2262		8.0 - 9.9 min

#	RT [min]	Area	Int. Type	I	S/N	Chromatogram	Max. m/z	FWHM [min]	Group
168	9.23	125433.5	MolFeature	13703	20.4		353.2297		8.0 - 9.9 min
169	9.25	15337.4	MolFeature	2722	9.2		331.2475		8.0 - 9.9 min
170	9.29	6346.4	MolFeature	1011	5.3		311.2211		8.0 - 9.9 min
171	9.30	18504.9	MolFeature	3198	9.9		351.214		8.0 - 9.9 min
172	9.33	23167.1	MolFeature	4243	47.1		197.1168		8.0 - 9.9 min
173	9.33	28087.8	MolFeature	5983	66.5		237.1097		8.0 - 9.9 min
174	9.34	24686.1	MolFeature	1998	5.1		868.4506		8.0 - 9.9 min
175	9.35	17896.4	MolFeature	2932	11.2		538.2122		8.0 - 9.9 min
176	9.35	17637.4	MolFeature	3163	10.6		516.23		8.0 - 9.9 min
177	9.41	18275.4	MolFeature	3215	10.8		331.2472		8.0 - 9.9 min
178	9.55	28192.4	MolFeature	1554	6.8		427.2662		8.0 - 9.9 min
179	9.68	17987.6	MolFeature	922	5		541.3936		8.0 - 9.9 min
180	9.68	28324.4	MolFeature	1471	4.5		558.4202		8.0 - 9.9 min
181	9.69	16330.1	MolFeature	856	4.2		563.3756		8.0 - 9.9 min
182	9.74	32123.2	MolFeature	3401	10.8		313.237		8.0 - 9.9 min
183	9.82	28777.1	MolFeature	3074	9.6		882.4666		8.0 - 9.9 min
184	9.87	11891332	MS(n)	2542638	754.3	MS(n): TIC +All MS	530.2465	0.07	8.0 - 9.9 min
185	9.87	13183.8	MolFeature	937	10.4		1086.4659		8.0 - 9.9 min
186	9.87	15959.4	MolFeature	732	8.1		1143.4389		8.0 - 9.9 min
187	9.88	8415.3	MolFeature	585	3		570.2295		8.0 - 9.9 min
188	9.88	2433472.5	Chromatogram	503863	680.2	BPC +All MS	530.2465	0.08	8.0 - 9.9 min
189	9.88	408079.7	MolFeature	64119	207		532.2457		8.0 - 9.9 min
190	9.88	1648251	MolFeature	170802	14.3		1076.5137		8.0 - 9.9 min
191	9.88	9156.7	MolFeature	824	9.2		590.2795		8.0 - 9.9 min
192	9.88	3211522.5	MolFeature	482877	487.3		530.2465		8.0 - 9.9 min
193	9.88	870544.7	MolFeature	71070	42.7		1081.4701		8.0 - 9.9 min
194	9.88	289109.2	MolFeature	19146	212.7		1610.7153		8.0 - 9.9 min
195	9.88	13608.2	MolFeature	971	3.7		1099.4431		8.0 - 9.9 min
196	9.88	1050164.1	MolFeature	116093	195.9		547.2729		8.0 - 9.9 min
197	9.88	131251.4	MolFeature	14578	50.9		549.2718		8.0 - 9.9 min
198	9.88	106197	MolFeature	10563	44		554.2273		8.0 - 9.9 min
199	9.88	857523.8	MolFeature	83148	148.5		552.2281		8.0 - 9.9 min
200	9.89	20951.5	MolFeature	1883	11.5		568.2026		8.0 - 9.9 min
201	9.89	27870.3	MolFeature	2164	4.7		1097.4421		8.0 - 9.9 min
202	9.91	10652.1	MolFeature	1052	4.5		794.3451		8.0 - 9.9 min
203	9.91	5023.1	MolFeature	372	3.2		862.4418		8.0 - 9.9 min
204	9.96	21054.5	MolFeature	1303	3.4		1141.4265		8.0 - 9.9 min
205	10.15	6349.9	MolFeature	375	4.2		802.3478		10.0 - 11.9 min
206	10.16	7598.1	MolFeature	392	8.7		1221.4813		10.0 - 11.9 min
207	10.18	2434439	MS(n)	610774	178.2	MS(n): TIC +All MS	530.2466	0.06	10.0 - 11.9 min
208	10.18	492936.7	Chromatogram	125541	168.2	BPC +All MS	530.2466	0.06	10.0 - 11.9 min
209	10.18	172864.8	MolFeature	16608	369.1		1059.4868		10.0 - 11.9 min
210	10.18	226799.7	MolFeature	27095	16.3		1081.4684		10.0 - 11.9 min

#	RT [min]	Area	Int. Type	I	S/N	Chromatogram	Max. m/z	FWHM [min]	Group
211	10.18	84022.7	MolFeature	14989	48.4		532.2454		10.0 - 11.9 min
212	10.18	678231.4	MolFeature	118721	119.8		530.2465		10.0 - 11.9 min
213	10.18	49467.8	MolFeature	6643	27.7		554.228		10.0 - 11.9 min
214	10.18	355083.7	MolFeature	58269	98.3		547.273		10.0 - 11.9 min
215	10.18	16449.8	MolFeature	1062	23.6		1097.4424		10.0 - 11.9 min
216	10.18	374522.1	MolFeature	53882	96.2		552.2285		10.0 - 11.9 min
217	10.19	50845.1	MolFeature	7355	25.7		549.2722		10.0 - 11.9 min
218	10.19	19426.3	MolFeature	2023	22.5		1149.4538		10.0 - 11.9 min
219	10.20	4609.9	MolFeature	383	4.3		794.3422		10.0 - 11.9 min
220	10.21	28175.1	MolFeature	3523	3.2		509.2757		10.0 - 11.9 min
221	10.55	5267.6	MolFeature	277	6.2		882.4695		10.0 - 11.9 min
222	10.55	8753.2	MolFeature	495	3.6		599.4354		10.0 - 11.9 min
223	10.56	13879.1	MolFeature	700	3.2		616.4619		10.0 - 11.9 min
224	10.58	24060.8	MolFeature	2113	12.1		353.2296		10.0 - 11.9 min
225	10.58	25544.3	MolFeature	2105	10.2		331.2476		10.0 - 11.9 min
226	10.63	12997.7	MolFeature	2349	26.1		328.2117		10.0 - 11.9 min
227	10.73	5867.6	MolFeature	201	4.5		806.5797		10.0 - 11.9 min
228	10.77	318729.2	MS(n)	116545	28.2	MS(n): TIC +All MS	274.2743	0.05	10.0 - 11.9 min
229	10.77	299229.3	Chromatogram	80943	107.2	BPC +All MS	274.2743	0.06	10.0 - 11.9 min
230	10.78	17717.3	MolFeature	2805	31.2		296.2561		10.0 - 11.9 min
231	10.78	408324.2	MolFeature	76291	362.6		274.2743		10.0 - 11.9 min
232	10.86	7076.2	MolFeature	1411	7.7		385.2583		10.0 - 11.9 min
233	10.89	39939.8	MolFeature	8463	6		230.2477		10.0 - 11.9 min
234	10.91	6510.9	MolFeature	849	9.4		333.1665		10.0 - 11.9 min
235	10.93	125807.6	Chromatogram	36454	46.1	BPC +All MS	318.3003	0.06	10.0 - 11.9 min
236	10.93	15642.5	MolFeature	2460	9.7		340.283		10.0 - 11.9 min
237	10.94	183772.3	MolFeature	33500	13.7		318.3004		10.0 - 11.9 min
238	10.99	16908.4	MolFeature	1646	5.4		334.296		10.0 - 11.9 min
239	11.01	36600.8	MolFeature	3354	25.7		362.3264		10.0 - 11.9 min
240	11.02	40052.3	MolFeature	4650	18		290.2692		10.0 - 11.9 min
241	11.02	6446.6	MolFeature	474	5.3		406.3519		10.0 - 11.9 min
242	11.03	21287	MolFeature	1718	8.5		607.1809		10.0 - 11.9 min
243	11.03	11885.6	MolFeature	795	3		377.1945		10.0 - 11.9 min
244	11.03	7400.7	MolFeature	635	7.1		439.1389		10.0 - 11.9 min
245	11.03	26305.1	MolFeature	1696	6		251.1254		10.0 - 11.9 min
246	11.03	16368.8	MolFeature	1504	6.2		329.0054		10.0 - 11.9 min
247	11.04	16091.1	MolFeature	1613	3.4		327.0081		10.0 - 11.9 min
248	11.04	3616.1	MolFeature	405	4.5		461.1196		10.0 - 11.9 min
249	11.05	14899.2	MolFeature	1537	3.6		197.1173		10.0 - 11.9 min
250	11.05	13137.5	MolFeature	895	6.1		771.4861		10.0 - 11.9 min
251	11.08	6097.1	MolFeature	570	3.8		409.1746		10.0 - 11.9 min
252	11.08	8482.2	MolFeature	998	3.5		267.1955		10.0 - 11.9 min
253	11.09	31587.2	MolFeature	4251	7.9		387.1931		10.0 - 11.9 min
#	RT [min]	Area	Int. Type	I	S/N	Chromatogram	Max. m/z	FWHM [min]	Group

254	11.10	22906.4	MolFeature	835	4.3		749.5103		10.0 - 11.9 min
255	11.11	59427.2	MolFeature	3033	8.4		835.5363		10.0 - 11.9 min
256	11.11	12014.4	MolFeature	455	3.8		864.6197		10.0 - 11.9 min
257	11.11	5436.6	MolFeature	416	4.6		582.2768		10.0 - 11.9 min
258	11.12	5733.4	MolFeature	688	7.1		869.5793		10.0 - 11.9 min
259	11.12	19000.9	MolFeature	2065	11.3		544.4055		10.0 - 11.9 min
260	11.12	19547.5	MolFeature	2536	4		272.2582		10.0 - 11.9 min
261	11.12	15539.3	MolFeature	866	4.2		331.2479		10.0 - 11.9 min
262	11.12	13682.4	MolFeature	1544	5.5		432.2376		10.0 - 11.9 min
263	11.12	19620.8	MolFeature	2628	7.9		500.3789		10.0 - 11.9 min
264	11.13	8568.8	MolFeature	1237	4.6		549.3598		10.0 - 11.9 min
265	11.13	13607.7	MolFeature	1351	3.5		505.3356		10.0 - 11.9 min
266	11.14	15654.2	MolFeature	2475	4.6		417.2817		10.0 - 11.9 min
267	11.14	25353.1	MolFeature	3549	25.7		415.2117		10.0 - 11.9 min
268	11.14	21054.7	MolFeature	3375	10.8		373.2571		10.0 - 11.9 min
269	11.14	11296.2	MolFeature	2081	8.2		387.273		10.0 - 11.9 min
270	11.14	10481.2	MolFeature	1283	6.3		351.2643		10.0 - 11.9 min
271	11.14	12551.3	MolFeature	2221	24.7		399.0514		10.0 - 11.9 min
272	11.14	37026	MolFeature	6201	10.9		397.0534		10.0 - 11.9 min
273	11.14	27207.3	MolFeature	2492	7.6		239.1619		10.0 - 11.9 min
274	11.14	9663.3	MolFeature	1316	3.7		307.2472		10.0 - 11.9 min
275	11.14	25817.4	MolFeature	2751	11.7		329.2296		10.0 - 11.9 min
276	11.15	11538.5	MolFeature	1091	4		437.1939		10.0 - 11.9 min
277	11.15	9090.3	MolFeature	1334	6.4		285.204		10.0 - 11.9 min
278	11.16	6460.8	MolFeature	844	4.2		409.2548		10.0 - 11.9 min
279	11.17	37514	MolFeature	1839	9.2		265.1051		10.0 - 11.9 min
280	11.17	12447.5	MolFeature	1476	4.8		249.1106		10.0 - 11.9 min
281	11.17	23217.4	MolFeature	3232	25.4		515.2636		10.0 - 11.9 min
282	11.17	84499.4	MolFeature	2965	7.8		575.5022		10.0 - 11.9 min
283	11.17	45227.5	MolFeature	1579	5.4		397.382		10.0 - 11.9 min
284	11.18	16616.6	MolFeature	1234	6.8		363.2118		10.0 - 11.9 min
285	11.18	14893.5	MolFeature	928	3.3		593.4061		10.0 - 11.9 min
286	11.19	106096.6	MolFeature	8880	22.9		589.1699		10.0 - 11.9 min
287	11.19	12812.8	MolFeature	1370	6.5		341.3054		10.0 - 11.9 min
288	11.19	17511.1	MolFeature	2121	7.2		359.3152		10.0 - 11.9 min
289	11.19	15234.4	MolFeature	2967	10.3		383.2067		10.0 - 11.9 min
290	11.19	7510.7	MolFeature	439	9.8		597.3541		10.0 - 11.9 min
291	11.20	30390	MolFeature	3635	6.9		456.2974		10.0 - 11.9 min
292	11.20	34831.4	MolFeature	5260	58.4		461.2506		10.0 - 11.9 min
293	11.20	7047.3	MolFeature	723	8		663.3419		10.0 - 11.9 min
294	11.21	200779.2	MolFeature	5319	16.6		593.5128		10.0 - 11.9 min
295	11.21	13700.6	MolFeature	350	4.2		845.5392		10.0 - 11.9 min
296	11.21	45875.7	MolFeature	1767	5.5		768.5982		10.0 - 11.9 min
297	11.21	30600.4	MolFeature	1109	5.2		335.2189		10.0 - 11.9 min
**#**	**RT [min]**	**Area**	**Int. Type**	**I**	**S/N**	**Chromatogram**	**Max. m/z**	**FWHM [min]**	**Group**
298	11.21	15010.1	MolFeature	294	3.4		1079.7671		10.0 - 11.9 min

299	11.21	22493.2	MolFeature	3380	4.9		611.1516		10.0 - 11.9 min
300	11.21	148740.3	MolFeature	3505	12		695.464		10.0 - 11.9 min
301	11.21	10564.3	MolFeature	758	4.2		857.5144		10.0 - 11.9 min
302	11.22	56677.9	MolFeature	3992	15.5		753.4759		10.0 - 11.9 min
303	11.22	57097.7	MolFeature	2286	8.6		697.4791		10.0 - 11.9 min
304	11.22	1024764.8	MS(n)	836428	65.9	MS(n): TIC +All MS	589.1697	0.05	10.0 - 11.9 min
305	11.22	17231	MolFeature	572	3.3		951.6042		10.0 - 11.9 min
306	11.22	8775.8	MolFeature	309	3.2		878.5762		10.0 - 11.9 min
307	11.22	33717.7	MolFeature	5784	11.2		292.1074		10.0 - 11.9 min
308	11.22	15623.8	MolFeature	2325	9		284.1419		10.0 - 11.9 min
309	11.23	6056.5	MolFeature	572	3.7		308.2951		10.0 - 11.9 min
310	11.23	55817.5	MolFeature	9756	8.5		296.2223		10.0 - 11.9 min
311	11.23	33192.2	MolFeature	779	3.2		853.663		10.0 - 11.9 min
312	11.23	6857	MolFeature	1102	8.8		223.1327		10.0 - 11.9 min
313	11.23	10181.2	MolFeature	541	4		967.6366		10.0 - 11.9 min
314	11.24	18570.2	MolFeature	885	5.1		333.2019		10.0 - 11.9 min
315	11.24	35790.5	MolFeature	2156	8.4		757.506		10.0 - 11.9 min
316	11.24	60805.3	MolFeature	668	4.8		1043.6985		10.0 - 11.9 min
317	11.24	6468.1	MolFeature	469	10.4		937.5599		10.0 - 11.9 min
318	11.24	11083	MolFeature	860	5.8		581.3688		10.0 - 11.9 min
319	11.24	6623.2	MolFeature	782	4.3		927.6215		10.0 - 11.9 min
320	11.25	6655.7	MolFeature	481	4.6		922.6674		10.0 - 11.9 min
321	11.25	9813.1	MolFeature	1492	6.3		419.2036		10.0 - 11.9 min
322	11.25	7841.2	MolFeature	979	7.4		732.5415		10.0 - 11.9 min
323	11.25	64511	MolFeature	1033	3.3		715.5086		10.0 - 11.9 min
324	11.26	51308.5	MolFeature	2717	5.4		997.7247		10.0 - 11.9 min
325	11.26	15061.3	MolFeature	1725	7.4		737.4996		10.0 - 11.9 min
326	11.26	24066.4	MolFeature	2610	9.9		399.3924		10.0 - 11.9 min
327	11.26	202195.3	MolFeature	8875	12.5		615.4949		10.0 - 11.9 min
328	11.26	30148.3	MolFeature	4470	26		288.2545		10.0 - 11.9 min
329	11.27	116706.6	MolFeature	10568	28.9		200.2009		10.0 - 11.9 min
330	11.27	20850.5	MolFeature	712	5.9		1023.7671		10.0 - 11.9 min
331	11.27	50656.5	MolFeature	6583	23.3		299.2581		10.0 - 11.9 min
332	11.27	33181.1	MolFeature	603	3.8		862.6424		10.0 - 11.9 min
333	11.27	7424.2	MolFeature	893	5.4		1001.6982		10.0 - 11.9 min
334	11.28	115996.9	MolFeature	3737	6.3		313.2729		10.0 - 11.9 min
335	11.28	17504.3	MolFeature	1402	5.2		931.7534		10.0 - 11.9 min
336	11.28	14429.8	MolFeature	2761	13.8		384.2898		10.0 - 11.9 min
337	11.28	15933.6	MolFeature	2506	11.5		347.1835		10.0 - 11.9 min
338	11.28	37521.2	MolFeature	5820	29.4		281.2475		10.0 - 11.9 min
339	11.28	335749.1	MolFeature	7943	19.7		610.5396		10.0 - 11.9 min
340	11.28	27399.9	MolFeature	4905	8		317.2686		10.0 - 11.9 min
**#**	**RT [min]**	**Area**	**Int. Type**	**I**	**S/N**	**Chromatogram**	**Max. m/z**	**FWHM [min]**	**Group**
341	11.29	17706.2	MolFeature	714	3.1		755.4843		10.0 - 11.9 min
342	11.29	12841.8	MolFeature	2129	8.5		331.1881		10.0 - 11.9 min

343	11.30	42433.8	MolFeature	2871	6.4		509.3563		10.0 - 11.9 min
344	11.30	55673.6	MolFeature	6522	27.5		305.1083		10.0 - 11.9 min
345	11.30	32881.8	MolFeature	1146	3.3		381.3716		10.0 - 11.9 min
346	11.30	28728.5	MolFeature	5695	20.8		167.0339		10.0 - 11.9 min
347	11.30	102447.4	MolFeature	7162	27.9		734.5406		10.0 - 11.9 min
348	11.30	11992	MolFeature	386	4.3		563.3842		10.0 - 11.9 min
349	11.31	185413.5	MolFeature	26714	101.5		739.4961		10.0 - 11.9 min
350	11.31	19204.5	MolFeature	1961	8.5		699.5025		10.0 - 11.9 min
351	11.31	99453	MolFeature	13623	46.7		503.2616		10.0 - 11.9 min
352	11.32	6114.6	MolFeature	308	6.8		581.9026		10.0 - 11.9 min
353	11.33	20774.3	MolFeature	4097	18		356.2427		10.0 - 11.9 min
354	11.33	15535.7	MolFeature	835	3.7		625.3367		10.0 - 11.9 min
355	11.35	8853.6	MolFeature	494	4		559.8886		10.0 - 11.9 min
356	11.35	14963.8	MolFeature	1216	5.9		474.4152		10.0 - 11.9 min
357	11.36	18778.5	MolFeature	1864	13		479.3702		10.0 - 11.9 min
358	11.36	22231.5	MolFeature	3739	9.5		488.2418		10.0 - 11.9 min
359	11.37	11796.1	MolFeature	548	4.9		485.8538		10.0 - 11.9 min
360	11.37	9143.7	MolFeature	495	3.3		953.6764		10.0 - 11.9 min
361	11.37	56272.5	MolFeature	7711	20.2		339.2194		10.0 - 11.9 min
362	11.38	22591.5	MolFeature	3002	9.4		272.2219		10.0 - 11.9 min
363	11.38	657050.1	Chromatogram	267563	325.9	BPC +All MS	457.2775	0.04	10.0 - 11.9 min
364	11.38	1496287.8	MS(n)	1193541	124.9	MS(n): TIC +All MS	457.2776	0.06	10.0 - 11.9 min
365	11.39	10810.8	MolFeature	511	3.9		948.7187		10.0 - 11.9 min
366	11.39	514282.4	MolFeature	112511	139		452.3219		10.0 - 11.9 min
367	11.39	32466.9	MolFeature	1200	4.6		568.4905		10.0 - 11.9 min
368	11.39	26895.1	MolFeature	867	4		277.1791		10.0 - 11.9 min
369	11.39	1202221.3	MolFeature	204369	414.8		457.2775		10.0 - 11.9 min
370	11.39	31494.1	MolFeature	6227	29.8		299.1615		10.0 - 11.9 min
371	11.40	11381.1	MolFeature	1015	5.3		790.5867		10.0 - 11.9 min
372	11.40	25604	MolFeature	4937	42		267.1577		10.0 - 11.9 min
373	11.40	30169.5	MolFeature	845	3.3		317.1962		10.0 - 11.9 min
374	11.41	237271.1	MolFeature	10103	21		685.435		10.0 - 11.9 min
375	11.41	6020	MolFeature	1390	9.7		235.1696		10.0 - 11.9 min
376	11.41	41493.8	MolFeature	995	4.4		748.5571		10.0 - 11.9 min
377	11.41	16341.2	MolFeature	965	4.5		455.3198		10.0 - 11.9 min
378	11.42	12924.2	MolFeature	584	5.7		791.4756		10.0 - 11.9 min
379	11.42	195090.6	MolFeature	10098	10.7		641.5105		10.0 - 11.9 min
380	11.43	547660.5	MolFeature	13872	30.1		663.4527		10.0 - 11.9 min
381	11.43	9527.5	MolFeature	473	10.5		593.1397		10.0 - 11.9 min
382	11.43	187347.4	MolFeature	32920	125.1		739.496		10.0 - 11.9 min
383	11.43	568908.4	MolFeature	14244	30.9		680.4792		10.0 - 11.9 min
**#**	**RT [min]**	**Area**	**Int. Type**	**I**	**S/N**	**Chromatogram**	**Max. m/z**	**FWHM [min]**	**Group**
384	11.44	102497.8	MolFeature	3019	9.7		726.5868		10.0 - 11.9 min
385	11.44	11499	MolFeature	634	14.1		515.8631		10.0 - 11.9 min
386	11.44	82038.5	MolFeature	11077	5.2		717.5131		10.0 - 11.9 min

387	11.46	17327.9	MolFeature	800	5.5		1083.7005		10.0 - 11.9 min
388	11.46	18275.3	MolFeature	2281	16.5		943.6167		10.0 - 11.9 min
389	11.46	18997.4	MolFeature	4640	18		609.3377		10.0 - 11.9 min
390	11.46	3996.6	MolFeature	963	4.2		604.3859		10.0 - 11.9 min
391	11.46	15038.8	MolFeature	1380	10		904.6923		10.0 - 11.9 min
392	11.47	10896.3	MolFeature	1068	4.1		330.2983		10.0 - 11.9 min
393	11.47	31463.5	MolFeature	9059	32.7		399.2501		10.0 - 11.9 min
394	11.47	49045.6	MolFeature	11267	101.5		259.1905		10.0 - 11.9 min
395	11.47	8639	MolFeature	904	5.8		760.222		10.0 - 11.9 min
396	11.47	17951.8	MolFeature	716	4.9		704.5119		10.0 - 11.9 min
397	11.47	120602	MolFeature	25640	96.8		281.1722		10.0 - 11.9 min
398	11.47	61290	MolFeature	11836	5		559.3244		10.0 - 11.9 min
399	11.48	33924.1	MolFeature	6111	29.1		421.2322		10.0 - 11.9 min
400	11.48	168045.6	MolFeature	7703	19.5		801.5497		10.0 - 11.9 min
401	11.48	30650.8	MolFeature	3051	8.9		963.5866		10.0 - 11.9 min
402	11.48	118657.5	MolFeature	6721	15.4		617.5113		10.0 - 11.9 min
403	11.49	30908.9	MolFeature	808	6.2				10.0 - 11.9 min
404	11.49	34391.6	MolFeature	8969	34.9		243.1354		10.0 - 11.9 min
405	11.49	297445.8	MolFeature	70789	786.5		579.2925		10.0 - 11.9 min
406	11.49	13289.9	MolFeature	709	3.5		779.566		10.0 - 11.9 min
407	11.49	14654.5	MolFeature	2979	8.7		221.1531		10.0 - 11.9 min
408	11.49	55644.4	MolFeature	517	3.2		920.7047		10.0 - 11.9 min
409	11.49	30961.6	MolFeature	8113	48		317.1151		10.0 - 11.9 min
410	11.49	119702.7	MolFeature	4741	13.4		796.5913		10.0 - 11.9 min
411	11.49	451364.9	MolFeature	97816	273.6		301.1411		10.0 - 11.9 min
412	11.49	177738.5	MolFeature	49318	344.2		205.0861		10.0 - 11.9 min
413	11.50	129005.4	MolFeature	15767	14.5		282.2791		10.0 - 11.9 min
414	11.50	339652.6	MolFeature	46136	189.7		388.3934		10.0 - 11.9 min
415	11.50	47815.8	MolFeature	5006	21.9		671.4489		10.0 - 11.9 min
416	11.51	104754	MolFeature	7516	6.2		633.4122		10.0 - 11.9 min
417	11.51	7569	MolFeature	838	5		577.3921		10.0 - 11.9 min
418	11.52	736027.4	MolFeature	104578	219.3		691.4173		10.0 - 11.9 min
419	11.52	14776.3	MolFeature	1562	5.8		528.4091		10.0 - 11.9 min
420	11.52	7407.5	MolFeature	1240	5.6		533.3654		10.0 - 11.9 min
421	11.52	541678.9	MolFeature	71201	220.5		279.1593		10.0 - 11.9 min
422	11.53	36580.4	MolFeature	1632	6		752.6015		10.0 - 11.9 min
423	11.53	15640	MolFeature	1839	11.2		484.3823		10.0 - 11.9 min
424	11.53	7866.8	MolFeature	1247	7.1		489.3391		10.0 - 11.9 min
425	11.53	199038.9	MolFeature	39989	146.6		302.305		10.0 - 11.9 min
426	11.53	479310.6	MolFeature	70458	232.3		149.0235		10.0 - 11.9 min
427	11.53	4649.1	MolFeature	652	5.6		1053.7297		10.0 - 11.9 min
**#**	**RT [min]**	**Area**	**Int. Type**	**I**	**S/N**	**Chromatogram**	**Max. m/z**	**FWHM [min]**	**Group**
428	11.53	22032.5	MolFeature	4916	36.4		318.2998		10.0 - 11.9 min
429	11.53	22982.9	MolFeature	2586	6.7		431.2457		10.0 - 11.9 min
430	11.54	112011.2	MolFeature	18142	66.2		167.0341		10.0 - 11.9 min
431	11.54	245444	MolFeature	46775	36.6		408.3102		10.0 - 11.9 min

432	11.54	2758735.5	Chromatogram	710650	927.2	BPC +All MS	803.5429	0.06	10.0 - 11.9 min
433	11.54	151885	MolFeature	16971	6.2		819.516		10.0 - 11.9 min
434	11.54	256517.6	MolFeature	43359	229.5		261.1486		10.0 - 11.9 min
435	11.54	3839697	MolFeature	566437	1144.3		391.284		10.0 - 11.9 min
436	11.54	277821.7	MolFeature	51058	185.8		113.1327		10.0 - 11.9 min
437	11.54	433281.8	MolFeature	48386	141.8		798.5873		10.0 - 11.9 min
438	11.54	14563.1	MolFeature	1274	3.8		860.6626		10.0 - 11.9 min
439	11.54	167209.3	MolFeature	25199	18.4		429.2397		10.0 - 11.9 min
440	11.54	4573864	MolFeature	685744	3.4		803.5429		10.0 - 11.9 min
441	11.54	17240668	MS(n)	3848534	927.4	MS(n): TIC +All MS	803.5429	0.08	10.0 - 11.9 min
442	11.54	152984.2	MolFeature	11445	95.7		1075.712		10.0 - 11.9 min
443	11.54	36679.1	MolFeature	7676	5.5		346.3313		10.0 - 11.9 min
444	11.54	20695	MolFeature	2334	4.4		394.2939		10.0 - 11.9 min
445	11.54	19710.4	MolFeature	1094	4.3		416.2767		10.0 - 11.9 min
446	11.54	37666.7	MolFeature	5692	126.5		648.5547		10.0 - 11.9 min
447	11.55	10518	MolFeature	1096	8.1		1070.756		10.0 - 11.9 min
448	11.55	2394733.5	MolFeature	282996	513.8		413.2659		10.0 - 11.9 min
449	11.55	18538.1	MolFeature	1678	6.3		1005.7697		10.0 - 11.9 min
450	11.55	37545.1	MolFeature	8684	9.8		258.279		10.0 - 11.9 min
451	11.56	70304.2	MolFeature	4946	21.4		1031.787		10.0 - 11.9 min
452	11.56	34950.6	MolFeature	4422	20		709.4995		10.0 - 11.9 min
453	11.56	18274.4	MolFeature	1696	6.5		297.2435		10.0 - 11.9 min
454	11.56	21442.2	MolFeature	1613	5.6		319.2249		10.0 - 11.9 min
455	11.56	8397.2	MolFeature	638	3.1		450.3558		10.0 - 11.9 min
456	11.56	7113	MolFeature	584	3.7		405.2582		10.0 - 11.9 min
457	11.56	6592.7	MolFeature	873	4.5		987.6909		10.0 - 11.9 min
458	11.57	15043.3	MolFeature	2577	14		401.2857		10.0 - 11.9 min
459	11.57	23043.3	MolFeature	5189	19.9		257.1511		10.0 - 11.9 min
460	11.57	36280.7	MolFeature	5390	16.6		251.1646		10.0 - 11.9 min
461	11.57	24559.7	MolFeature	2478	55.1		952.6372		10.0 - 11.9 min
462	11.57	13041	MolFeature	1695	8.4		252.195		10.0 - 11.9 min
463	11.57	10626.5	MolFeature	864	8.6		455.4555		10.0 - 11.9 min
464	11.57	26287.8	MolFeature	3885	10.1		357.2605		10.0 - 11.9 min
465	11.58	5816.4	MolFeature	1250	4		352.3052		10.0 - 11.9 min
466	11.58	24363.9	MolFeature	1021	6.9		865.6168		10.0 - 11.9 min
467	11.58	36879.1	MolFeature	7266	35.7		562.3612		10.0 - 11.9 min
468	11.58	25635.7	MolFeature	3123	9.6		750.5995		10.0 - 11.9 min
469	11.58	719925.5	MolFeature	121699	273.2		540.3791		10.0 - 11.9 min
470	11.59	20924.8	MolFeature	1289	4.8		581.4359		10.0 - 11.9 min
**#**	**RT [min]**	**Area**	**Int. Type**	**I**	**S/N**	**Chromatogram**	**Max. m/z**	**FWHM [min]**	**Group**
471	11.59	50126.5	MolFeature	4672	17.7		310.3103		10.0 - 11.9 min
472	11.59	20915.3	MolFeature	1110	5.6		827.6284		10.0 - 11.9 min
473	11.59	36333.5	MolFeature	6318	24.8		425.2151		10.0 - 11.9 min
474	11.59	1081946.9	MolFeature	78879	222.7		338.3415		10.0 - 11.9 min
475	11.60	12168.4	MolFeature	654	5.6		594.9134		10.0 - 11.9 min

476	11.60	25266.1	MolFeature	3505	11		719.4823		10.0 - 11.9 min
477	11.60	34157.3	MolFeature	1367	6		1000.7825		10.0 - 11.9 min
478	11.60	79414.3	MolFeature	6122	24.5		228.2322		10.0 - 11.9 min
479	11.60	54250	MolFeature	9059	36.3		329.2081		10.0 - 11.9 min
480	11.60	27278.7	MolFeature	2002	7.4		825.6197		10.0 - 11.9 min
481	11.60	74518	MolFeature	11395	48.8		324.253		10.0 - 11.9 min
482	11.60	60406.5	MolFeature	7883	28.4		554.3935		10.0 - 11.9 min
483	11.60	184717.5	MolFeature	8385	17.4		685.436		10.0 - 11.9 min
484	11.60	15086.3	MolFeature	3244	14.5		307.2264		10.0 - 11.9 min
485	11.60	87757.2	MolFeature	4179	14.6		395.3656		10.0 - 11.9 min
486	11.61	36716.7	MolFeature	2925	8.6		675.6746		10.0 - 11.9 min
487	11.61	12476.8	MolFeature	2500	6.4		291.253		10.0 - 11.9 min
488	11.61	6757.7	MolFeature	1356	4.8		365.2117		10.0 - 11.9 min
489	11.61	48729.1	MolFeature	1977	6.3		597.4068		10.0 - 11.9 min
490	11.61	11081.4	MolFeature	2369	8		612.4582		10.0 - 11.9 min
491	11.61	13219.3	MolFeature	875	4.5		443.24		10.0 - 11.9 min
492	11.61	10542.5	MolFeature	957	7.7		833.6247		10.0 - 11.9 min
493	11.62	58641.5	MolFeature	8580	23.5		200.2007		10.0 - 11.9 min
494	11.62	5472.1	MolFeature	304	6.8		919.545		10.0 - 11.9 min
495	11.62	33180.4	MolFeature	3618	16.4		435.3436		10.0 - 11.9 min
496	11.62	11416.3	MolFeature	1647	8.3		222.1821		10.0 - 11.9 min
497	11.63	99903.1	MolFeature	14112	53.6		739.4953		10.0 - 11.9 min
498	11.63	56853.1	MolFeature	1215	5.2		754.5825		10.0 - 11.9 min
499	11.63	19972.8	MolFeature	1516	14.1		898.6087		10.0 - 11.9 min
500	11.63	28774.2	MolFeature	5552	24.8		269.2087		10.0 - 11.9 min
501	11.63	47443.5	MolFeature	5309	18.2		413.3618		10.0 - 11.9 min
502	11.63	30641.2	MolFeature	1302	9.1		903.5673		10.0 - 11.9 min
503	11.63	3656.8	MolFeature	572	5.3		931.656		10.0 - 11.9 min
504	11.63	34945.9	MolFeature	3479	20.9		854.5831		10.0 - 11.9 min
505	11.64	73978.2	MolFeature	3924	14.3		815.5064		10.0 - 11.9 min
506	11.64	20150.6	MolFeature	2416	13.3		859.5391		10.0 - 11.9 min
507	11.64	6333	MolFeature	498	5.5		421.3681		10.0 - 11.9 min
508	11.64	13273.6	MolFeature	1099	7.5		821.5954		10.0 - 11.9 min
509	11.65	19754.3	MolFeature	1918	8		313.3574		10.0 - 11.9 min
510	11.65	31204.7	MolFeature	1819	11.5		816.6388		10.0 - 11.9 min
511	11.65	37416.8	MolFeature	5881	30.7		810.5568		10.0 - 11.9 min
512	11.65	2387049	MolFeature	77660	102.3		676.4356		10.0 - 11.9 min
513	11.66	25245.4	MolFeature	1485	7		536.3741		10.0 - 11.9 min
514	11.66	66954.1	MolFeature	9412	59.9		766.5305		10.0 - 11.9 min
**#**	**RT [min]**	**Area**	**Int. Type**	**I**	**S/N**	**Chromatogram**	**Max. m/z**	**FWHM [min]**	**Group**
515	11.66	26042.3	MolFeature	750	4.2		572.8993		10.0 - 11.9 min
516	11.67	83939	MolFeature	9271	23.6		727.4598		10.0 - 11.9 min
517	11.67	154700.1	MolFeature	14277	78.3		722.5035		10.0 - 11.9 min
518	11.67	39468	MolFeature	3653	19.5		439.3787		10.0 - 11.9 min
519	11.67	5092.2	MolFeature	432	4.3		1065.7052		10.0 - 11.9 min
520	11.68	64539.3	MolFeature	11460	29.4		256.2632		10.0 - 11.9 min

521	11.68	97033.5	MolFeature	10824	5.1		683.4346		10.0 - 11.9 min
522	11.68	32711.4	MolFeature	1770	4.9		594.5134		10.0 - 11.9 min
523	11.68	20245.5	MolFeature	2522	13.2		461.3594		10.0 - 11.9 min
524	11.68	2953078	MS(n)	1267596	151.2	MS(n): TIC +All MS	676.4338	0.1	10.0 - 11.9 min
525	11.68	25172.6	MolFeature	1627	4.4		560.5021		10.0 - 11.9 min
526	11.69	79941.3	MolFeature	2408	5.6		583.4821		10.0 - 11.9 min
527	11.69	77260.6	MolFeature	11123	45.5		639.4077		10.0 - 11.9 min
528	11.69	119793	MolFeature	19134	62.2		634.4524		10.0 - 11.9 min
529	11.69	150915.4	MolFeature	21968	116.3		359.3152		10.0 - 11.9 min
530	11.69	72742.5	MolFeature	7188	159.7		739.6042		10.0 - 11.9 min
531	11.69	87783.4	MolFeature	722	3.3		955.7082		10.0 - 11.9 min
532	11.69	126842	MolFeature	14696	9.9		595.3828		10.0 - 11.9 min
533	11.69	21593.4	MolFeature	3307	8.6		287.2213		10.0 - 11.9 min
534	11.70	90347.2	MolFeature	4205	8.4		997.7237		10.0 - 11.9 min
535	11.70	110977.5	MolFeature	17239	44.6		381.2973		10.0 - 11.9 min
536	11.70	8063.1	MolFeature	1103	12.3		267.2681		10.0 - 11.9 min
537	11.70	165056.7	MolFeature	20375	72.5		590.4265		10.0 - 11.9 min
538	11.70	20074.1	MolFeature	1657	11.3		1037.7453		10.0 - 11.9 min
539	11.70	19163.4	MolFeature	1431	12.4		911.6276		10.0 - 11.9 min
540	11.70	10691.7	MolFeature	839	3.8		533.3816		10.0 - 11.9 min
541	11.70	10224.7	MolFeature	526	3.5		793.5574		10.0 - 11.9 min
542	11.70	17662.2	MolFeature	915	3.7		577.4829		10.0 - 11.9 min
543	11.70	7952.8	MolFeature	975	5.8		906.6699		10.0 - 11.9 min
544	11.71	17921.9	MolFeature	1091	4.4		630.4773		10.0 - 11.9 min
545	11.71	18113.6	MolFeature	1594	3.8		573.4071		10.0 - 11.9 min
546	11.71	127376	MolFeature	13389	28.9		551.3558		10.0 - 11.9 min
547	11.72	103510.5	MolFeature	16602	56.8		546.3994		10.0 - 11.9 min
548	11.72	13091.9	MolFeature	772	3.6		425.3415		10.0 - 11.9 min
549	11.72	14302.8	MolFeature	1908	10.6		321.2404		10.0 - 11.9 min
550	11.73	79317.1	MolFeature	11156	35.2		502.3734		10.0 - 11.9 min
551	11.73	51185.8	MolFeature	4739	6		506.4405		10.0 - 11.9 min
552	11.73	68631	MolFeature	13664	4.6		463.3025		10.0 - 11.9 min
553	11.73	25570	MolFeature	2432	9.3		703.533		10.0 - 11.9 min
554	11.73	59426.7	MolFeature	7181	48.9		511.3963		10.0 - 11.9 min
555	11.73	96468.7	MolFeature	8139	36.8		725.5163		10.0 - 11.9 min
556	11.73	30855.5	MolFeature	2558	15.2		772.6046		10.0 - 11.9 min
557	11.74	58195.8	MolFeature	10894	27.1		419.2767		10.0 - 11.9 min
**#**	**RT [min]**	**Area**	**Int. Type**	**I**	**S/N**	**Chromatogram**	**Max. m/z**	**FWHM [min]**	**Group**
558	11.74	17351.7	MolFeature	2532	5.6		370.2956		10.0 - 11.9 min
559	11.74	29630.9	MolFeature	6065	54.8		375.2506		10.0 - 11.9 min
560	11.75	6832.6	MolFeature	612	4.6		706.4777		10.0 - 11.9 min
561	11.75	7945.8	MolFeature	1224	5.8		331.2243		10.0 - 11.9 min
562	11.75	1602920.5	MolFeature	25317	26.9		641.5103		10.0 - 11.9 min
563	11.76	368528.2	MolFeature	4428	9.2		601.516		10.0 - 11.9 min
564	11.76	48045.6	MolFeature	2775	12.2		498.3989		10.0 - 11.9 min

565	11.76	8086.3	MolFeature	962	6.5		550.8857		10.0 - 11.9 min
566	11.77	72538.9	MolFeature	2355	9.8		803.5765		10.0 - 11.9 min
567	11.78	18364.5	MolFeature	651	14.5		852.5711		10.0 - 11.9 min
568	11.79	1845105.6	MolFeature	27074	34.4		636.5551		10.0 - 11.9 min
569	11.80	17532.3	MolFeature	1705	5.2		410.347		10.0 - 11.9 min
570	11.80	31930.3	MolFeature	1575	6.9		610.1841		10.0 - 11.9 min
571	11.80	49497	MolFeature	1422	8.1		798.6392		10.0 - 11.9 min
572	11.81	449752.1	MS(n)	860129	29.2	MS(n): TIC +All MS	282.2792	0.09	10.0 - 11.9 min
573	11.81	20810.8	MolFeature	3641	20		355.245		10.0 - 11.9 min
574	11.81	8870.5	MolFeature	1440	16		350.2907		10.0 - 11.9 min
575	11.82	18495.3	MolFeature	635	3.4		612.1814		10.0 - 11.9 min
576	11.82	9543.6	MolFeature	1702	6.6		366.3208		10.0 - 11.9 min
577	11.83	9318.3	MolFeature	483	4.1		1034.7546		10.0 - 11.9 min
578	11.83	31119.5	MolFeature	4053	24.8		371.2762		10.0 - 11.9 min
579	11.83	77339.9	MolFeature	1868	8.2		570.5433		10.0 - 11.9 min
580	11.83	94973.4	MolFeature	3508	15.6		764.5614		10.0 - 11.9 min
581	11.83	339505	MolFeature	12876	48.7		742.582		10.0 - 11.9 min
582	11.84	28513.6	MolFeature	925	4.7		1029.7481		10.0 - 11.9 min
583	11.84	27980.6	MolFeature	1087	4.3		529.3753		10.0 - 11.9 min
584	11.84	11214.6	MolFeature	2315	3.5		411.2717		10.0 - 11.9 min
585	11.84	18213.8	MolFeature	606	3.7		609.4483		10.0 - 11.9 min
586	11.84	62676.2	MolFeature	2990	17.5		776.554		10.0 - 11.9 min
587	11.85	139792.3	MolFeature	18477	17		282.2791		10.0 - 11.9 min
588	11.85	37995.7	MolFeature	2621	13.4		728.5849		10.0 - 11.9 min
589	11.85	1055824.6	MolFeature	14183	25.2		619.528		10.0 - 11.9 min
590	11.85	21389.6	MolFeature	831	6.5		304.2598		10.0 - 11.9 min
591	11.85	64449.6	MolFeature	2010	10.5		468.4759		10.0 - 11.9 min
592	11.86	17638.4	MolFeature	1513	8.9		588.4101		10.0 - 11.9 min
593	11.86	56903.7	MolFeature	3264	8.1		532.4556		10.0 - 11.9 min
594	11.86	71635.4	MolFeature	5597	16.5		537.411		10.0 - 11.9 min
595	11.86	19202.3	MolFeature	2217	10.1		435.3438		10.0 - 11.9 min
596	11.87	25937.7	MolFeature	1684	9.5		733.5442		10.0 - 11.9 min
597	11.87	13410.9	MolFeature	717	4.3		593.3671		10.0 - 11.9 min
598	11.87	20083.3	MolFeature	5834	17.8		327.2498		10.0 - 11.9 min
599	11.87	75328.3	MolFeature	3330	20.5		429.2584		10.0 - 11.9 min
600	11.87	99229.8	MolFeature	3790	11.5		427.2453		10.0 - 11.9 min
**#**	**RT [min]**	**Area**	**Int. Type**	**I**	**S/N**	**Chromatogram**	**Max. m/z**	**FWHM [min]**	**Group**
601	11.88	16126	MolFeature	1988	5.4		595.4312		10.0 - 11.9 min
602	11.88	25418.2	MolFeature	1961	11.1		1051.7246		10.0 - 11.9 min
603	11.88	9457.4	MolFeature	580	4.8		1046.7667		10.0 - 11.9 min
604	11.90	14478.3	MolFeature	1297	5.5		820.5981		10.0 - 11.9 min
605	11.90	16588.9	MolFeature	3599	13.7		283.2233		10.0 - 11.9 min
606	11.90	31419.1	MolFeature	802	3.6		533.383		10.0 - 11.9 min
607	11.90	54169.8	MolFeature	2769	17.3		770.5777		10.0 - 11.9 min
608	11.90	21684.6	MolFeature	736	3.9		507.3568		10.0 - 11.9 min

#	RT [min]	Area	Int. Type	I	S/N	Chromatogram	Max. m/z	FWHM [min]	Group
609	11.91	74413.8	MolFeature	1065	4.6		673.4978		10.0 - 11.9 min
610	11.91	45321.3	MolFeature	1974	7.1		591.4933		10.0 - 11.9 min
611	11.92	12465.3	MolFeature	984	3.5		781.5289		10.0 - 11.9 min
612	11.93	5516.5	MolFeature	770	6.2		417.2601		10.0 - 11.9 min
613	11.93	3998.2	MolFeature	451	5		582.8958		10.0 - 11.9 min
614	11.93	11388.8	MolFeature	1342	4.2		693.4754		10.0 - 11.9 min
615	11.93	8859.9	MolFeature	581	3		560.8806		10.0 - 11.9 min
616	11.94	143576.4	MolFeature	1840	7		990.7537		10.0 - 11.9 min
617	11.94	37224.5	MolFeature	1945	10.5		649.4506		10.0 - 11.9 min
618	11.95	33300.3	MolFeature	427	3.3		874.6342		10.0 - 11.9 min
619	11.96	79058.9	MolFeature	3370	10.8		688.5186		10.0 - 11.9 min
620	11.96	79553.6	MolFeature	14872	29.2		330.3364		10.0 - 11.9 min
621	11.96	11872.1	MolFeature	929	7		538.8675		10.0 - 11.9 min
622	11.97	13495.1	MolFeature	1171	6.4		516.8543		10.0 - 11.9 min
623	11.98	80263.9	MolFeature	12561	32.3		256.2634		10.0 - 11.9 min
624	11.98	26850	MolFeature	2471	8.3		684.5592		10.0 - 11.9 min
625	11.98	184016	MS(n)	848222	24.3	MS(n): TIC +All MS	330.3363	0.02	10.0 - 11.9 min
626	11.98	14493	MolFeature	729	3.7				10.0 - 11.9 min
627	11.99	14263.4	MolFeature	531	4.8		951.6601		10.0 - 11.9 min
628	11.99	53773	MolFeature	4037	17.2		556.441		10.0 - 11.9 min
629	11.99	19655.7	MolFeature	1267	5.9		494.8397		10.0 - 11.9 min
630	11.99	101508.2	MolFeature	2370	6.3		992.7592		10.0 - 11.9 min
631	12.00	13178.9	MolFeature	654	3.3		1013.6775		12.0 - 13.9 min
632	12.00	55735.7	MolFeature	1930	5.7		561.3953		12.0 - 13.9 min
633	12.00	132249.8	MolFeature	5192	10.4		997.7128		12.0 - 13.9 min
634	12.00	21010.5	MolFeature	1250	7.7		946.7031		12.0 - 13.9 min
635	12.00	12920.5	MolFeature	1017	5.5		472.8264		12.0 - 13.9 min
636	12.00	48288.7	MolFeature	6280	28.4		725.5159		12.0 - 13.9 min
637	12.01	12498.3	MolFeature	426	4.1		922.6737		12.0 - 13.9 min
638	12.02	37443	MolFeature	2884	12.3		517.3691		12.0 - 13.9 min
639	12.02	107458.7	MolFeature	13422	51.7		284.2946		12.0 - 13.9 min
640	12.02	15994.6	MolFeature	591	3.4		669.5003		12.0 - 13.9 min
641	12.02	3993.5	MolFeature	463	3.6		883.6017		12.0 - 13.9 min
642	12.02	6035.3	MolFeature	492	5.5		351.2492		12.0 - 13.9 min
643	12.02	58190.1	MolFeature	6072	33.1		512.4146		12.0 - 13.9 min
**#**	**RT [min]**	**Area**	**Int. Type**	**I**	**S/N**	**Chromatogram**	**Max. m/z**	**FWHM [min]**	**Group**
644	12.03	26411	MolFeature	4374	27		420.3311		12.0 - 13.9 min
645	12.03	61837.6	MolFeature	11210	77.1		425.2869		12.0 - 13.9 min
646	12.04	28669.2	MolFeature	803	3.4		878.6382		12.0 - 13.9 min
647	12.04	131869.4	MolFeature	1715	5.9		848.7082		12.0 - 13.9 min
648	12.04	255745.9	MS(n)	887739	33.8	MS(n): TIC +All MS	425.2872	0.04	12.0 - 13.9 min
649	12.04	12900.5	MolFeature	1878	10.2		369.2608		12.0 - 13.9 min
650	12.04	6362.9	MolFeature	630	3.2		364.3047		12.0 - 13.9 min
651	12.05	12596.3	MolFeature	917	6.2		839.5727		12.0 - 13.9 min
652	12.05	31719.8	MolFeature	4942	3.9		356.352		12.0 - 13.9 min

653	12.05	25287.1	MolFeature	608	3.3		611.9139		12.0 - 13.9 min
654	12.05	52944.7	MolFeature	5232	25.8		473.3436		12.0 - 13.9 min
655	12.06	35149.7	MolFeature	1621	9.5		834.613		12.0 - 13.9 min
656	12.06	3743.1	MolFeature	509	3.3		372.3453		12.0 - 13.9 min
657	12.06	67312.3	MolFeature	4041	8.1		568.4899		12.0 - 13.9 min
658	12.06	28380.8	MolFeature	2248	11.8		790.5866		12.0 - 13.9 min
659	12.07	59385.7	MolFeature	3411	14.8		551.4652		12.0 - 13.9 min
660	12.07	67292.6	MolFeature	4806	19.2		295.2264		12.0 - 13.9 min
661	12.07	112752.1	MolFeature	7486	8.1		573.4457		12.0 - 13.9 min
662	12.07	75034.9	MolFeature	3940	24.6		702.5356		12.0 - 13.9 min
663	12.08	55601	MolFeature	1709	6.9		751.5171		12.0 - 13.9 min
664	12.08	17971.3	MolFeature	1461	15.4		902.6752		12.0 - 13.9 min
665	12.08	39740.7	MolFeature	1158	7.1		567.8881		12.0 - 13.9 min
666	12.08	52385.4	MolFeature	3184	21.7		746.5622		12.0 - 13.9 min
667	12.08	49476.2	MolFeature	1371	4		983.7147		12.0 - 13.9 min
668	12.09	77114.5	MolFeature	1650	7.5		709.502		12.0 - 13.9 min
669	12.09	10114.2	MolFeature	678	4.8		856.6352		12.0 - 13.9 min
670	12.09	214270.8	MS(n)	891910	32.7	MS(n): TIC +All MS	467.3674	0.04	12.0 - 13.9 min
671	12.09	46833.5	MolFeature	3413	12.8		736.5309		12.0 - 13.9 min
672	12.09	22555.1	MolFeature	637	3.2		565.4094		12.0 - 13.9 min
673	12.09	50553.2	MolFeature	1769	9.1		667.4891		12.0 - 13.9 min
674	12.09	27031.8	MolFeature	5617	27.9		429.3176		12.0 - 13.9 min
675	12.09	36290.7	MolFeature	3327	11.5		424.3621		12.0 - 13.9 min
676	12.10	147627.4	MolFeature	10903	14.3		714.5497		12.0 - 13.9 min
677	12.10	6889.2	MolFeature	1305	10		325.2343		12.0 - 13.9 min
678	12.10	203413.5	MolFeature	24856	138		467.3697		12.0 - 13.9 min
679	12.10	21976	MolFeature	1246	6.7		545.8739		12.0 - 13.9 min
680	12.10	69954.1	MolFeature	2960	8.4		796.5907		12.0 - 13.9 min
681	12.10	120142.4	MolFeature	14645	27.4		462.4145		12.0 - 13.9 min
682	12.10	43712.1	MolFeature	2508	13.5		707.4903		12.0 - 13.9 min
683	12.11	32654.6	MolFeature	891	4		521.3856		12.0 - 13.9 min
684	12.11	16451.3	MolFeature	1190	4.2		445.3841		12.0 - 13.9 min
685	12.12	88735.9	MolFeature	3491	14		801.5521		12.0 - 13.9 min
686	12.12	43923	MolFeature	1266	4.2		311.2549		12.0 - 13.9 min
**#**	**RT [min]**	**Area**	**Int. Type**	**I**	**S/N**	**Chromatogram**	**Max. m/z**	**FWHM [min]**	**Group**
687	12.12	83156.9	MolFeature	3705	8		663.4617		12.0 - 13.9 min
688	12.12	153989.5	MolFeature	5677	26.5		658.5071		12.0 - 13.9 min
689	12.13	12697.4	MolFeature	2747	16		380.3371		12.0 - 13.9 min
690	12.13	16246.7	MolFeature	2784	6.5		402.3571		12.0 - 13.9 min
691	12.13	14262.7	MolFeature	1159	3.5		645.4909		12.0 - 13.9 min
692	12.13	38950.5	MolFeature	6794	28.3		385.2915		12.0 - 13.9 min
693	12.13	10118.9	MolFeature	994	5.6		812.609		12.0 - 13.9 min
694	12.14	63982.9	MolFeature	1573	8.3		523.8612		12.0 - 13.9 min
695	12.14	101682	MolFeature	3707	16.5		619.4372		12.0 - 13.9 min
696	12.15	21347.5	MolFeature	4831	12.4		256.2632		12.0 - 13.9 min

697	12.15	435411.1	MS(n)	909401	35.7	MS(n): TIC +All MS	326.3777	0.07	12.0 - 13.9 min
698	12.15	40905.8	MolFeature	1098	6.5		501.8486		12.0 - 13.9 min
699	12.15	87809	Chromatogram	61241	57	BPC +All MS	326.3778	0.03	12.0 - 13.9 min
700	12.15	137696.9	MolFeature	7237	24.9		570.4566		12.0 - 13.9 min
701	12.15	14336.3	MolFeature	985	5.2		479.8351		12.0 - 13.9 min
702	12.16	84533.7	MolFeature	4210	15.5		575.4115		12.0 - 13.9 min
703	12.16	288735.3	MolFeature	48439	199.8		326.3778		12.0 - 13.9 min
704	12.16	41429.3	MolFeature	853	6.8		892.6524		12.0 - 13.9 min
705	12.17	11461.7	MolFeature	752	8.2		863.6075		12.0 - 13.9 min
706	12.17	97303.4	MolFeature	639	4		936.7407		12.0 - 13.9 min
707	12.17	66286.4	MolFeature	876	3		941.7014		12.0 - 13.9 min
708	12.17	16799.1	MolFeature	1729	10.1		858.6483		12.0 - 13.9 min
709	12.17	152071.5	MolFeature	6965	27.5		526.4292		12.0 - 13.9 min
710	12.18	8891.3	MolFeature	685	3.1		457.8208		12.0 - 13.9 min
711	12.18	10495.4	MolFeature	462	3.3		897.6108		12.0 - 13.9 min
712	12.18	16019.1	MolFeature	1494	5.7		310.3105		12.0 - 13.9 min
713	12.18	132229.9	MolFeature	3973	9.6		531.3834		12.0 - 13.9 min
714	12.19	31530	MolFeature	6710	37.2		341.2659		12.0 - 13.9 min
715	12.19	57486.4	MolFeature	3195	18.1		1051.7249		12.0 - 13.9 min
716	12.19	14889.6	MolFeature	1983	14		319.2836		12.0 - 13.9 min
717	12.19	9419.5	MolFeature	830	5.2		768.5834		12.0 - 13.9 min
718	12.19	19677.9	MolFeature	669	4.2		559.3701		12.0 - 13.9 min
719	12.19	73705.2	MolFeature	4126	19.8		482.4039		12.0 - 13.9 min
720	12.20	71034.1	MolFeature	3642	17.9		487.359		12.0 - 13.9 min
721	12.20	37726.8	MolFeature	1000	5.1		1029.7462		12.0 - 13.9 min
722	12.20	8103.5	MolFeature	767	3.7		392.3874		12.0 - 13.9 min
723	12.20	34015.9	MolFeature	1472	7.6		520.4337		12.0 - 13.9 min
724	12.20	52648.2	MolFeature	1655	8.7		399.3072		12.0 - 13.9 min
725	12.21	57669.8	MolFeature	3357	15.1		443.3329		12.0 - 13.9 min
726	12.21	37071.1	MolFeature	2059	12.2		438.3781		12.0 - 13.9 min
727	12.21	15230	MolFeature	1004	3.8		471.4028		12.0 - 13.9 min
728	12.22	216461.3	MolFeature	4029	9.9		995.7144		12.0 - 13.9 min
729	12.22	17313.8	MolFeature	584	3.5		596.9089		12.0 - 13.9 min
**#**	**RT [min]**	**Area**	**Int. Type**	**I**	**S/N**	**Chromatogram**	**Max. m/z**	**FWHM [min]**	**Group**
730	12.23	179078.7	MolFeature	1465	4.1		804.6006		12.0 - 13.9 min
731	12.23	10202.8	MolFeature	628	3		699.5043		12.0 - 13.9 min
732	12.23	224257.9	MolFeature	17697	71		493.3854		12.0 - 13.9 min
733	12.23	408249.2	MS(n)	879740	29.9	MS(n): TIC +All MS	669.3438	0.04	12.0 - 13.9 min
734	12.23	164133	MolFeature	13245	56.1		488.4328		12.0 - 13.9 min
735	12.23	24016.1	MolFeature	4964	19.2		297.2394		12.0 - 13.9 min
736	12.23	12348.9	MolFeature	2688	21		275.258		12.0 - 13.9 min
737	12.23	53379.1	MolFeature	1427	6.4		355.2813		12.0 - 13.9 min
738	12.23	185516.8	MolFeature	1529	7.4		760.5754		12.0 - 13.9 min
739	12.25	88313.4	MolFeature	15674	55.5		669.344		12.0 - 13.9 min
740	12.25	11547.2	MolFeature	673	5.6		580.4554		12.0 - 13.9 min

741	12.26	40526.9	MolFeature	1531	7.9		745.466		12.0 - 13.9 min
742	12.26	18426.3	MolFeature	2073	6.6		703.4733		12.0 - 13.9 min
743	12.26	16937.5	MolFeature	1430	14.2		536.1644		12.0 - 13.9 min
744	12.26	22075	MolFeature	1748	13.1		814.6232		12.0 - 13.9 min
745	12.27	7653.7	MolFeature	443	3.8		538.1641		12.0 - 13.9 min
746	12.27	102051.6	MolFeature	3753	14.8		723.4829		12.0 - 13.9 min
747	12.28	22910.8	MolFeature	1212	5.6		934.6464		12.0 - 13.9 min
748	12.28	64840.9	MolFeature	2550	11.3		939.6014		12.0 - 13.9 min
749	12.28	148526.6	MolFeature	10575	25.5		630.5073		12.0 - 13.9 min
750	12.28	22913.6	MolFeature	749	4		530.8699		12.0 - 13.9 min
751	12.28	143237.6	MolFeature	10281	27.4		635.4632		12.0 - 13.9 min
752	12.29	10287.7	MolFeature	652	3.3		427.3026		12.0 - 13.9 min
753	12.29	35091.1	MolFeature	1404	4.8		383.2752		12.0 - 13.9 min
754	12.30	1541435.3	MolFeature	39613	52.2		676.4353		12.0 - 13.9 min
755	12.30	32767.2	MolFeature	936	4.6		552.8847		12.0 - 13.9 min
756	12.30	26450.5	MolFeature	2629	7.1		596.5028		12.0 - 13.9 min
757	12.32	6433.6	MolFeature	1048	5.5		439.3015		12.0 - 13.9 min
758	12.32	60019.3	MolFeature	1758	9.8		1011.6791		12.0 - 13.9 min
759	12.33	373527.3	Chromatogram	41647	35.9	BPC +All MS	676.4354	0.25	12.0 - 13.9 min
760	12.33	7887.3	MolFeature	941	6.2		378.322		12.0 - 13.9 min
761	12.33	56489.8	MolFeature	1850	7.4		577.4847		12.0 - 13.9 min
762	12.33	11129.5	MolFeature	1142	6.7		665.4928		12.0 - 13.9 min
763	12.33	88376.2	MolFeature	4991	13.9		599.4643		12.0 - 13.9 min
764	12.36	16626.4	MolFeature	930	3.4		534.4858		12.0 - 13.9 min
765	12.36	16660	MolFeature	1112	4.2		313.2724		12.0 - 13.9 min
766	12.37	113185.5	MolFeature	1411	4.5		436.4318		12.0 - 13.9 min
767	12.37	20936.9	MolFeature	1845	11.5		770.5983		12.0 - 13.9 min
768	12.38	28080.2	MolFeature	1427	7.1		1013.6771		12.0 - 13.9 min
769	12.38	12773.8	MolFeature	885	3.5		1015.7044		12.0 - 13.9 min
770	12.39	72022	MolFeature	2917	7.8		992.7473		12.0 - 13.9 min
771	12.39	63295	MolFeature	2204	9.4		589.4269		12.0 - 13.9 min
772	12.40	154818	MolFeature	7106	14.2		997.7032		12.0 - 13.9 min
**#**	**RT [min]**	**Area**	**Int. Type**	**I**	**S/N**	**Chromatogram**	**Max. m/z**	**FWHM [min]**	**Group**
773	12.40	61358.3	MolFeature	1584	6.4		721.502		12.0 - 13.9 min
774	12.40	111581.6	MolFeature	2730	11.1		552.4939		12.0 - 13.9 min
775	12.41	36778.6	MolFeature	930	6.7		823.5783		12.0 - 13.9 min
776	12.42	27553.4	MolFeature	2083	11		539.3699		12.0 - 13.9 min
777	12.42	247544.3	MolFeature	15775	55.7		517.3878		12.0 - 13.9 min
778	12.42	6875.3	MolFeature	1356	7.6		339.2487		12.0 - 13.9 min
779	12.42	64664.6	MolFeature	2587	9		672.5223		12.0 - 13.9 min
780	12.43	20149.3	MolFeature	755	3.1		1006.7436		12.0 - 13.9 min
781	12.44	150086.7	MolFeature	6543	13.4		632.5222		12.0 - 13.9 min
782	12.45	175188	MolFeature	6634	26.3		637.4787		12.0 - 13.9 min
783	12.45	101195.7	MolFeature	3486	13.5		628.4972		12.0 - 13.9 min
784	12.45	180275.8	MolFeature	5757	23.2		726.5843		12.0 - 13.9 min
785	12.45	33440.9	MolFeature	1182	4.9		312.3258		12.0 - 13.9 min

786	12.46	59855.5	MolFeature	1107	6.2		562.5169		12.0 - 13.9 min
787	12.47	70977.5	MolFeature	3206	11.4		584.4715		12.0 - 13.9 min
788	12.49	50475.5	MolFeature	1625	6.7		752.6012		12.0 - 13.9 min
789	12.49	29460.5	MolFeature	2041	5.6		545.4004		12.0 - 13.9 min
790	12.50	18049.3	MolFeature	1714	8.7		557.4352		12.0 - 13.9 min
791	12.50	112402.9	MolFeature	1325	5.9		748.5666		12.0 - 13.9 min
792	12.50	122739	MolFeature	1940	7		634.5361		12.0 - 13.9 min
793	12.51	88879.5	MolFeature	3666	10.9		540.4453		12.0 - 13.9 min
794	12.51	43583.1	MolFeature	2380	7.1		501.3763		12.0 - 13.9 min
795	12.52	37218.4	MolFeature	2948	6.6		496.4201		12.0 - 13.9 min
796	12.53	176887.4	MolFeature	11858	15.6		714.5488		12.0 - 13.9 min
797	12.53	87240.8	MolFeature	5063	19		736.5324		12.0 - 13.9 min
798	12.53	24075.9	MolFeature	899	3.7		647.4731		12.0 - 13.9 min
799	12.54	29885.7	MolFeature	1171	5.8		452.3957		12.0 - 13.9 min
800	12.54	102905.3	MolFeature	13744	93.3		639.3346		12.0 - 13.9 min
801	12.54	31476.9	MolFeature	2060	5.6		457.3484		12.0 - 13.9 min
802	12.54	87318.8	MolFeature	2203	7.2		575.4983		12.0 - 13.9 min
803	12.55	32035.3	MolFeature	930	3.3		775.5563		12.0 - 13.9 min
804	12.55	7216.3	MolFeature	845	3		414.3932		12.0 - 13.9 min
805	12.57	11541.9	MolFeature	1885	9.9		490.3369		12.0 - 13.9 min
806	12.57	26780.9	MolFeature	1521	5.3		965.7056		12.0 - 13.9 min
807	12.57	21970.5	MolFeature	1481	6.3		591.4179		12.0 - 13.9 min
808	12.58	56990.1	MolFeature	2443	6.3		608.5228		12.0 - 13.9 min
809	12.59	71553.1	MolFeature	3686	14		613.4781		12.0 - 13.9 min
810	12.59	22937.4	MolFeature	1499	3		391.2835		12.0 - 13.9 min
811	12.60	170400.7	MolFeature	1247	4.5		959.7152		12.0 - 13.9 min
812	12.60	55325.1	MolFeature	3180	5.8		413.2659		12.0 - 13.9 min
813	12.61	25870.5	MolFeature	1131	4.3		1005.7538		12.0 - 13.9 min
814	12.61	23193.8	MolFeature	720	3.7		829.6199		12.0 - 13.9 min
815	12.61	729019	MS(n)	796687	61	MS(n): TIC +All MS	353.2662	0.05	12.0 - 13.9 min
**#**	**RT [min]**	**Area**	**Int. Type**	**I**	**S/N**	**Chromatogram**	**Max. m/z**	**FWHM [min]**	**Group**
816	12.61	31852.2	MolFeature	1316	6.7		1029.7467		12.0 - 13.9 min
817	12.61	123245.5	MolFeature	16518	3.6		683.542		12.0 - 13.9 min
818	12.61	57351	MolFeature	3258	8		995.7144		12.0 - 13.9 min
819	12.61	80291.1	Chromatogram	33065	31	BPC +All MS	353.2662	0.04	12.0 - 13.9 min
820	12.61	86407.6	MolFeature	2961	7.2		639.4913		12.0 - 13.9 min
821	12.61	17162	MolFeature	3090	12.2		348.3102		12.0 - 13.9 min
822	12.61	43940.6	MolFeature	2099	5.9		338.3415		12.0 - 13.9 min
823	12.61	10176.2	MolFeature	1545	10.8		421.2528		12.0 - 13.9 min
824	12.62	16305.5	MolFeature	984	3.8		711.5349		12.0 - 13.9 min
825	12.62	45525.7	MolFeature	2267	12.8		1051.7236		12.0 - 13.9 min
826	12.62	143492.1	MolFeature	29669	114.8		353.2661		12.0 - 13.9 min
827	12.62	101487.6	MolFeature	23087	92.2		331.2836		12.0 - 13.9 min
828	12.62	36439.7	MolFeature	7592	28.4		313.2733		12.0 - 13.9 min
829	12.62	75326	MolFeature	3105	11.7		742.5802		12.0 - 13.9 min

#	RT [min]	Area	Int. Type	I	S/N	Chromatogram	Max. m/z	FWHM [min]	Group
830	12.63	10030.7	MolFeature	1633	11.5		306.2766		12.0 - 13.9 min
831	12.63	55427	MolFeature	7293	81		711.3542		12.0 - 13.9 min
832	12.63	17907.8	MolFeature	572	3.1		1027.7327		12.0 - 13.9 min
833	12.63	11973.5	MolFeature	606	3		827.6021		12.0 - 13.9 min
834	12.63	45295.3	MolFeature	9135	13.9		284.2944		12.0 - 13.9 min
835	12.64	46535.5	MolFeature	2504	11.6		465.3896		12.0 - 13.9 min
836	12.66	6513.5	MolFeature	523	3.1		813.6095		12.0 - 13.9 min
837	12.66	46483.7	MolFeature	5544	30.4		401.3617		12.0 - 13.9 min
838	12.66	169180.2	MolFeature	20165	87.5		423.3439		12.0 - 13.9 min
839	12.67	68668.5	MolFeature	8140	45.9		418.3883		12.0 - 13.9 min
840	12.69	22343.3	MolFeature	3080	12.6		453.319		12.0 - 13.9 min
841	12.71	29464.8	MolFeature	839	6.1		912.6258		12.0 - 13.9 min
842	12.71	3491	MolFeature	419	3.7		500.8437		12.0 - 13.9 min
843	12.71	50192.1	MolFeature	1626	8.6		410.4345		12.0 - 13.9 min
844	12.71	18711.1	MolFeature	2343	6		628.1937		12.0 - 13.9 min
845	12.72	13018.1	MolFeature	839	5.3		868.5994		12.0 - 13.9 min
846	12.73	14413.4	MolFeature	1507	5.1		513.409		12.0 - 13.9 min
847	12.73	14929.8	MolFeature	909	3.3		508.4554		12.0 - 13.9 min
848	12.75	120784.7	MolFeature	1230	4.7		978.743		12.0 - 13.9 min
849	12.77	30026.8	MolFeature	676	4.2		846.6187		12.0 - 13.9 min
850	12.78	106720.2	MolFeature	8436	32.2		444.4043		12.0 - 13.9 min
851	12.78	158798.4	MolFeature	14960	73		449.3596		12.0 - 13.9 min
852	12.78	72854.1	MolFeature	3855	13		427.3827		12.0 - 13.9 min
853	12.79	5272	MolFeature	465	4.1		325.2697		12.0 - 13.9 min
854	12.79	96018.3	MolFeature	2980	12.8		756.597		12.0 - 13.9 min
855	12.81	11679.6	MolFeature	717	3.2		571.4337		12.0 - 13.9 min
856	12.81	23470.6	MolFeature	656	5.6		609.9336		12.0 - 13.9 min
857	12.81	42238.1	MolFeature	1024	4.6		684.2012		12.0 - 13.9 min
858	12.81	10145.7	MolFeature	1446	4.3		540.4454		12.0 - 13.9 min
859	12.82	30517.1	MolFeature	531	3.3		686.1999		12.0 - 13.9 min
860	12.82	20030.9	MolFeature	631	6.2		916.6554		12.0 - 13.9 min
861	12.83	8414.3	MolFeature	972	4.6		310.3104		12.0 - 13.9 min
862	12.84	7237.9	MolFeature	1098	3.3		461.3013		12.0 - 13.9 min
863	12.84	9800	MolFeature	726	5.9		877.5854		12.0 - 13.9 min
864	12.85	15632.6	MolFeature	1031	7.4		461.4334		12.0 - 13.9 min
865	12.85	22501.4	MolFeature	1228	3.1		496.4687		12.0 - 13.9 min
866	12.85	13254.1	MolFeature	813	3.3		801.5833		12.0 - 13.9 min
867	12.85	20382.4	MolFeature	966	5.5		580.5268		12.0 - 13.9 min
868	12.85	18524.5	MolFeature	990	4		837.6625		12.0 - 13.9 min
869	12.85	77639.1	MolFeature	4760	16.9		479.4448		12.0 - 13.9 min
870	12.86	9203.2	MolFeature	857	5.8		872.63		12.0 - 13.9 min
871	12.87	67934.7	MolFeature	3974	11.5		501.4253		12.0 - 13.9 min
872	12.87	93581.4	MolFeature	1716	5.7		992.7461		12.0 - 13.9 min
873	12.87	9384.4	MolFeature	359	8		923.755		12.0 - 13.9 min
874	12.87	7114.7	MolFeature	737	3.7		1013.7018		12.0 - 13.9 min

875	12.88	77004.9	MolFeature	2318	7.3		719.4627		12.0 - 13.9 min
876	12.88	18182.3	MolFeature	1181	6.1		829.6125		12.0 - 13.9 min
877	12.89	35432.8	MolFeature	1656	5.5				12.0 - 13.9 min
878	12.89	9386.3	MolFeature	1584	4.6		983.7117		12.0 - 13.9 min
879	12.89	19722.9	MolFeature	1818	6.9		1005.7538		12.0 - 13.9 min
880	12.89	17887.3	MolFeature	1281	6.5		1029.7461		12.0 - 13.9 min
881	12.89	28037.7	MolFeature	1009	3.9		990.7522		12.0 - 13.9 min
882	12.89	12911.2	MolFeature	1172	5.4		985.7217		12.0 - 13.9 min
883	12.89	16124.6	MolFeature	3216	17.1		239.236		12.0 - 13.9 min
884	12.89	25654.7	MolFeature	694	3.3		1009.716		12.0 - 13.9 min
885	12.89	20371.6	MolFeature	2102	8.7		784.5811		12.0 - 13.9 min
886	12.89	29500.2	MolFeature	5102	19.6		257.2475		12.0 - 13.9 min
887	12.89	56023.8	MolFeature	4741	11.6		995.7123		12.0 - 13.9 min
888	12.89	620007.6	MS(n)	709842	56.4	MS(n): TIC +All MS	445.3305	0.05	12.0 - 13.9 min
889	12.89	13417.2	MolFeature	728	5.3		841.5871		12.0 - 13.9 min
890	12.89	18244.7	MolFeature	979	3.7		969.7318		12.0 - 13.9 min
891	12.89	21414.6	MolFeature	2432	8.4		965.7033		12.0 - 13.9 min
892	12.89	6405.2	MolFeature	512	3.2		869.6244		12.0 - 13.9 min
893	12.89	9853.7	MolFeature	882	4.9		1011.7168		12.0 - 13.9 min
894	12.89	93004.5	MolFeature	1236	3.8		693.4451		12.0 - 13.9 min
895	12.89	144001.3	MolFeature	1846	6.3		848.7077		12.0 - 13.9 min
896	12.90	7368.7	MolFeature	1133	5.5		981.6971		12.0 - 13.9 min
897	12.90	26836.6	MolFeature	1999	5.8		853.6618		12.0 - 13.9 min
898	12.90	14730.4	MolFeature	435	3.6		913.6326		12.0 - 13.9 min
899	12.90	18067.5	MolFeature	1100	6		1031.7661		12.0 - 13.9 min
900	12.90	10408.9	MolFeature	782	4.9		1027.7368		12.0 - 13.9 min
901	12.90	12736.1	MolFeature	617	4.8		971.7239		12.0 - 13.9 min
902	12.90	47072.3	MolFeature	3805	25.8		1037.7459		12.0 - 13.9 min
**#**	**RT [min]**	**Area**	**Int. Type**	**I**	**S/N**	**Chromatogram**	**Max. m/z**	**FWHM [min]**	**Group**
903	12.90	96566.9	MolFeature	666	3.4		973.7291		12.0 - 13.9 min
904	12.90	29807.6	MolFeature	767	5		895.6251		12.0 - 13.9 min
905	12.90	26027	MolFeature	1215	27		963.7013		12.0 - 13.9 min
906	12.91	14478.4	MolFeature	748	4.4		467.3132		12.0 - 13.9 min
907	12.91	283535.8	Chromatogram	44465	47.5	BPC +All MS	445.3303	0.14	12.0 - 13.9 min
908	12.91	36099.1	MolFeature	487	4.3		815.5819		12.0 - 13.9 min
909	12.91	11200.3	MolFeature	631	3.3		947.7011		12.0 - 13.9 min
910	12.91	11586.8	MolFeature	456	3.9		1019.736		12.0 - 13.9 min
911	12.91	42503.9	MolFeature	2954	5.4		413.2652		12.0 - 13.9 min
912	12.91	22769.7	MolFeature	979	4.2		1001.7248		12.0 - 13.9 min
913	12.91	26494.5	MolFeature	804	3.4		659.5069		12.0 - 13.9 min
914	12.92	11108.2	MolFeature	1586	4.3		740.5476		12.0 - 13.9 min
915	12.92	12754.5	MolFeature	535	3.6		857.6058		12.0 - 13.9 min
916	12.93	11138.3	MolFeature	603	4.9		1003.7366		12.0 - 13.9 min
917	12.93	632482	MolFeature	42641	37.3		445.3305		12.0 - 13.9 min
918	12.93	21030.9	MolFeature	1199	4.8		701.4936		12.0 - 13.9 min

919	12.95	16685.5	MolFeature	1718	10.2		405.3689		12.0 - 13.9 min
920	12.95	8100.5	MolFeature	515	3.2		953.7142		12.0 - 13.9 min
921	12.95	49767.1	MolFeature	3275	9.2		383.3869		12.0 - 13.9 min
922	12.96	24599.8	MolFeature	903	5.4		955.6885		12.0 - 13.9 min
923	12.98	13361.3	MolFeature	1189	5.9		469.3828		12.0 - 13.9 min
924	13.00	11623.9	MolFeature	624	3.6		945.7274		12.0 - 13.9 min
925	13.00	21404	MolFeature	709	4		556.5262		12.0 - 13.9 min
926	13.01	9608.1	MolFeature	744	4.5		399.2494		12.0 - 13.9 min
927	13.02	56091.2	MolFeature	7054	16.2		697.3757		12.0 - 13.9 min
928	13.03	19091	MolFeature	650	5.8		610.3118		12.0 - 13.9 min
929	13.03	129093.8	MolFeature	2528	13.6		550.6272		12.0 - 13.9 min
930	13.05	33374.5	MolFeature	1242	6.1		540.5329		12.0 - 13.9 min
931	13.05	94900.5	MolFeature	3083	9.6		423.3969		12.0 - 13.9 min
932	13.06	11312.8	MolFeature	923	5.6		379.3558		12.0 - 13.9 min
933	13.06	19100.1	MolFeature	678	5.5		1003.739		12.0 - 13.9 min
934	13.09	221738.6	MolFeature	3893	9.7		610.5379		12.0 - 13.9 min
935	13.09	79518.4	MolFeature	1067	6.8		760.22		12.0 - 13.9 min
936	13.09	22345.6	MolFeature	758	3.5		967.6394		12.0 - 13.9 min
937	13.09	24310.8	MolFeature	1026	3.2		436.4318		12.0 - 13.9 min
938	13.10	221291.3	MolFeature	9259	46.9		564.5336		12.0 - 13.9 min
939	13.10	101215.9	MolFeature	5157	13.7		635.4631		12.0 - 13.9 min
940	13.10	75084	Chromatogram	17100	12	BPC +All MS	429.3718	0.14	12.0 - 13.9 min
941	13.11	3251233.5	MS(n)	567213	64.2	MS(n): TIC +All MS	663.44	0.2	12.0 - 13.9 min
942	13.11	30401.1	MolFeature	4186	8.4		306.2761		12.0 - 13.9 min
943	13.11	87631.9	MolFeature	5332	9.1		630.5074		12.0 - 13.9 min
944	13.11	30317.9	MolFeature	1660	5		542.4202		12.0 - 13.9 min
945	13.11	12886.1	MolFeature	661	3.6		586.5139		12.0 - 13.9 min
**#**	**RT [min]**	**Area**	**Int. Type**	**I**	**S/N**	**Chromatogram**	**Max. m/z**	**FWHM [min]**	**Group**
946	13.11	12523.6	MolFeature	785	3.9		341.3403		12.0 - 13.9 min
947	13.11	46340.2	MolFeature	1364	5.1		977.7267		12.0 - 13.9 min
948	13.11	34213.8	MolFeature	1220	6		680.5924		12.0 - 13.9 min
949	13.11	127374.9	MolFeature	15398	23.5		284.2944		12.0 - 13.9 min
950	13.12	11036.9	MolFeature	1944	12.1		369.2958		12.0 - 13.9 min
951	13.12	55283.1	MolFeature	3241	12.3		613.4803		12.0 - 13.9 min
952	13.12	19238.6	MolFeature	909	4		764.5383		12.0 - 13.9 min
953	13.12	5008.4	MolFeature	237	5.3		484.4716		12.0 - 13.9 min
954	13.13	331331.3	MolFeature	13782	28.6		685.4341		12.0 - 13.9 min
955	13.13	309666	MolFeature	11771	25.5		680.4789		12.0 - 13.9 min
956	13.14	61025.7	MolFeature	1903	6		593.5106		12.0 - 13.9 min
957	13.14	54547.4	MolFeature	1182	3.8		717.4535		12.0 - 13.9 min
958	13.14	13849	MolFeature	540	4		597.4468		12.0 - 13.9 min
959	13.15	10812.7	MolFeature	708	3.6		1029.7351		12.0 - 13.9 min
960	13.15	10782.6	MolFeature	2035	22.6		367.2805		12.0 - 13.9 min
961	13.15	63484.6	MolFeature	1268	4.7		671.4652		12.0 - 13.9 min
962	13.16	28230.6	MolFeature	2052	3.7		413.2651		12.0 - 13.9 min

963	13.16	273091.9	MolFeature	13730	29.8		663.4464		12.0 - 13.9 min
964	13.17	23777.7	MolFeature	1844	3.7		391.2836		12.0 - 13.9 min
965	13.17	7033.8	MolFeature	621	4.4		494.4551		12.0 - 13.9 min
966	13.17	161050.8	MolFeature	2756	10.3		697.4798		12.0 - 13.9 min
967	13.17	62478.9	MolFeature	3202	10.1		395.3666		12.0 - 13.9 min
968	13.18	17045.9	MolFeature	1075	8		485.3604		12.0 - 13.9 min
969	13.18	50673.7	MolFeature	2185	7.5		695.4646		12.0 - 13.9 min
970	13.18	28500.3	MolFeature	1213	5		453.334		12.0 - 13.9 min
971	13.19	7812.3	MolFeature	810	5.7		310.3096		12.0 - 13.9 min
972	13.20	165612.5	MolFeature	13982	60.5		429.3719		12.0 - 13.9 min
973	13.21	24698.9	MolFeature	702	4.3		784.5087		12.0 - 13.9 min
974	13.21	11152.4	MolFeature	664	4		461.3615		12.0 - 13.9 min
975	13.22	18587.6	MolFeature	1572	6.3		701.4964		12.0 - 13.9 min
976	13.22	42556.1	MolFeature	2921	11.3		445.3562		12.0 - 13.9 min
977	13.23	27273.8	MolFeature	651	3.4		578.5171		12.0 - 13.9 min
978	13.23	43014.4	MolFeature	3286	10.7		575.5012		12.0 - 13.9 min
979	13.23	14426.9	MolFeature	1051	6.6		603.5324		12.0 - 13.9 min
980	13.24	9323.3	MolFeature	530	11.8		850.5465		12.0 - 13.9 min
981	13.24	7734.8	MolFeature	452	3.1		833.5218		12.0 - 13.9 min
982	13.25	25474.2	MolFeature	1327	6.7		854.573		12.0 - 13.9 min
983	13.26	13911.3	MolFeature	1120	8.9		880.5887		12.0 - 13.9 min
984	13.26	17738.8	MolFeature	1513	11.6		885.5459		12.0 - 13.9 min
985	13.26	38779.6	MolFeature	2787	8.4		837.5487		12.0 - 13.9 min
986	13.26	69611.7	MolFeature	5056	34.5		835.5331		12.0 - 13.9 min
987	13.26	22622.8	MolFeature	1628	10.8		863.5624		12.0 - 13.9 min
988	13.26	35724.5	MolFeature	2947	15		852.5595		12.0 - 13.9 min
989	13.26	53763.6	MolFeature	4127	22.8		857.5139		12.0 - 13.9 min
**#**	**RT [min]**	**Area**	**Int. Type**	**I**	**S/N**	**Chromatogram**	**Max. m/z**	**FWHM [min]**	**Group**
990	13.26	3199.6	MolFeature	271	6		907.5269		12.0 - 13.9 min
991	13.26	11915.3	MolFeature	831	4.7		861.5455		12.0 - 13.9 min
992	13.26	33740.5	MolFeature	2789	18.4		859.5294		12.0 - 13.9 min
993	13.34	7608.4	MolFeature	638	4.1		873.477		12.0 - 13.9 min
